# Creation of a gene expression portrait of depression and its application for identifying potential treatments

**DOI:** 10.1038/s41598-021-83348-0

**Published:** 2021-02-15

**Authors:** Stephen C. Gammie

**Affiliations:** Department of Integrative Biology, University of Wisconsin-Madison, Madison, USA

**Keywords:** Neuroscience, Diseases of the nervous system

## Abstract

Depression is a complex mental health disorder and the goal here was to identify a consistent underlying portrait of expression that ranks all genes from most to least dysregulated and indicates direction of change relative to controls. Using large-scale neural gene expression depression datasets, a combined portrait (for men and women) was created along with one for men and one for women only. The depressed brain was characterized by a “hypo” state, that included downregulation of activity-related genes, including EGR1, FOS, and ARC, and indications of a lower brain temperature and sleep-like state. MAP kinase and BDNF pathways were enriched with overlapping genes. Expression patterns suggested decreased signaling for GABA and for neuropeptides, CRH, SST, and CCK. GWAS depression genes were among depression portrait genes and common genes of interest included SPRY2 and PSEN2. The portraits were used with the drug repurposing approach of signature matching to identify treatments that could reverse depression gene expression patterns. Exercise was identified as the top treatment for depression for the combined and male portraits. Other non-traditional treatments that scored well were: curcumin, creatine, and albiflorin. Fluoxetine scored best among typical antidepressants. The creation of the portraits of depression provides new insights into the complex landscape of depression and a novel platform for evaluating and identifying potential new treatments.

## Introduction

Disorders of the nervous system in humans, such as depression, are complicated, difficult to study, and difficult to treat. One inherent complexity of human nervous system disorders is that a typical person has about 80+ billion neurons (and similar number of glial cells)^1^ and most of the more than 20,000 protein coding genes are expressed in the brain^2^. Further, thousands of genes can exhibit altered expression for a given disorder when analyzing large-scale gene expression patterns in the brain taken postmortem from disorder and control individuals^3,4^. For major depressive disorder (MDD), multiple large-scale gene expression studies have been performed^4–9^, but a question that remains is whether there is a consistent signature of upregulated and downregulated genes across brain regions relative to controls^10^. In support of a pattern, a recent study of a subset of MDD expression datasets identified a consistent expression pattern for genes in the MAP kinase pathway that were altered with depression^4^. The goal of the present study was to take advantage of numerous large-scale gene expression studies and bioinformatics approaches to (1) create a gene expression portrait of depression that ranks the most to least dysregulated genes along with the direction of change and (2) use the gene expression portraits and drug repurposing tools to identify potential treatments for depression.

First, I make use of 29 publicly available large-scale gene expression datasets to create a portrait of depression that models more completely the exceptionally complex gene expression landscape that underlies this disorder. The critical component of the portrait is that it emphasizes consistent findings while penalizing inconsistent findings such that core components of depression expression can emerge. Given that there are sex differences for most disorders, in addition to a combined portrait, separate portraits for men and women were also created. Each gene expression portrait contains a ranking of all genes from most to least dysregulated along with the direction of change relative to non-depressed controls. With these I examine the basis of depression using enrichment analysis and gene network analysis. I also evaluate the portraits in association with results from recent GWAS studies on depression and evaluate similarities and differences in the male and female depression profiles. Comparisons to a similar approach that emphasizes parameters differently^11^ is also provided.

Second, I use the gene expression portraits to identify potential treatments for depression. I use the drug repurposing approach of signature matching and the premise that if one knows the direction of change of gene expression in a disorder and one knows the direction of gene expression change produced by a treatment, then one can identify treatments that move expression of the genes in the disorder back to the normal state^12,13^. In other words, the treatment reverses the expression pattern of the disorder. This approach has been validated using multiple datasets of clinically effective treatments along with tissues sets from related disorders^14^ and has been used successfully to identify new treatments for a range of disorders^15–17^. However, a caveat is that for a given disorder it is not known whether a reversal of gene expression patterns would necessarily lead to treatment and the approach here for depression is meant to be exploratory. For this analysis, I only evaluated gene expression datasets that were performed examining effects of treatments in the brain or related tissue (e.g., neuronal stem cells) across a range of species. Together, I curated and evaluated over 200 gene expression treatment datasets and compared these with the three gene expression portraits of depression. I rank these treatments for potential reversal of depression expression patterns.

## Results

The full list for each portrait with a ranking of all genes from most to least dysregulated with depression along with information on direction of expression change (up in depression = positive sign, down in depression = negative sign) is provided in Supplementary Table 1. Supplementary Fig. 1 provides an overview of the creation of the portraits. Two approaches were used to evaluate how well the portraits provide a consistent biological signature of depression. In the first, I examined how well each portrait captured the patterns of the original individual datasets, including those used to create the portrait and those not. Using a rank rank hypergeometric approach, the combined portrait matched well both datasets used and not used in its creation (see Supplementary Figs. 2 and 3 and Supplementary Table 1 for details). The male and female portraits best matched male and female datasets, respectively.

**Figure 1:**
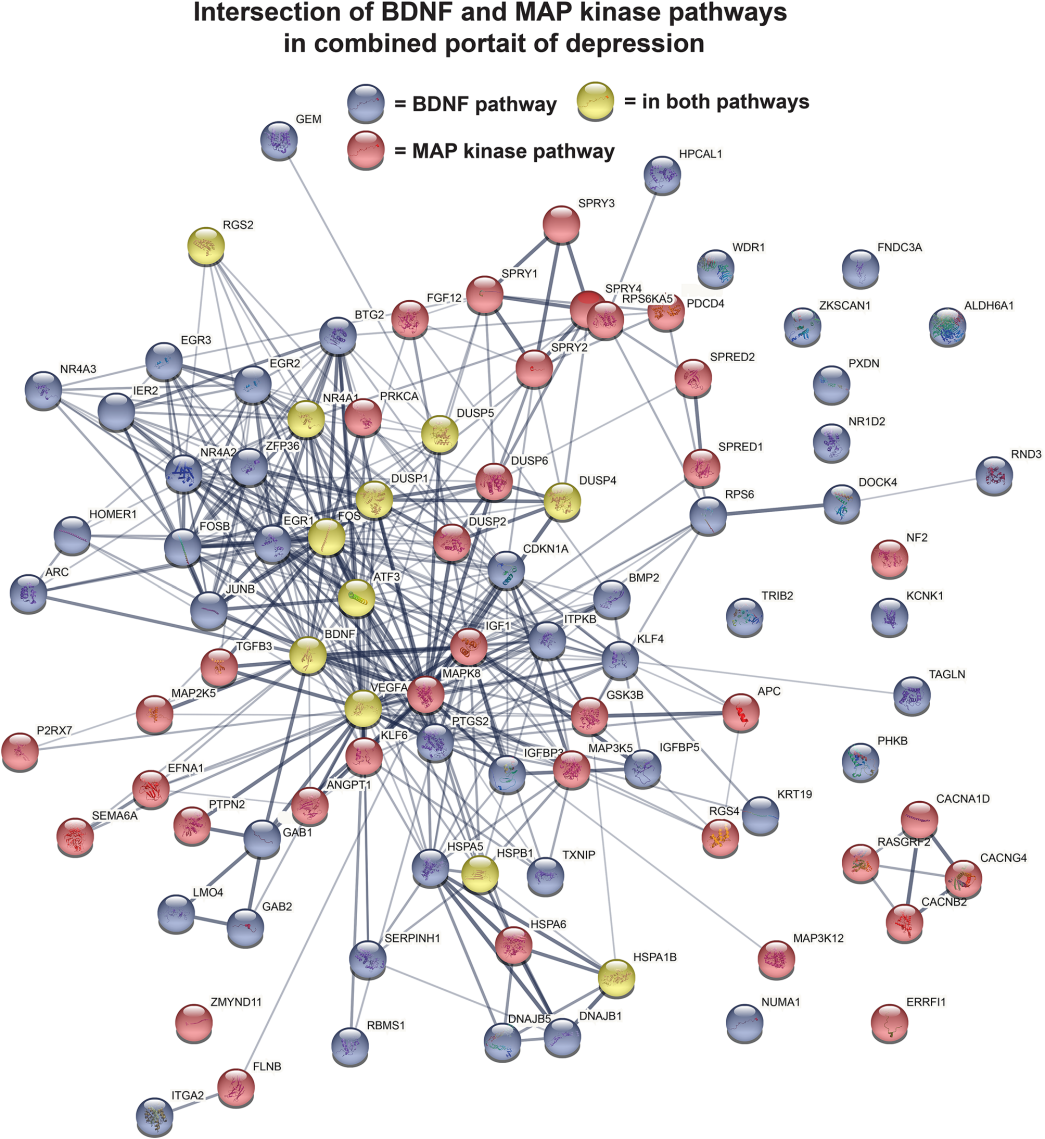
BDNF and MAP kinase pathways in combined portrait of depression. Genes enriched for BDNF signaling (blue circles) and MAP kinase pathway (red circles) were enriched among the top 1000 dysregulated genes in the combined portrait. Further, a subset of genes is in both pathways (yellow circles), including BDNF, DUSP1, DUSP4, DUSP5, FOS, MAP2K5, MAPK8, NR4A1, and two heatshock proteins. Plotting of gene interactions in STRING (version 11)^18^ highlights gene–gene interactions (lines).

**Figure 2:**
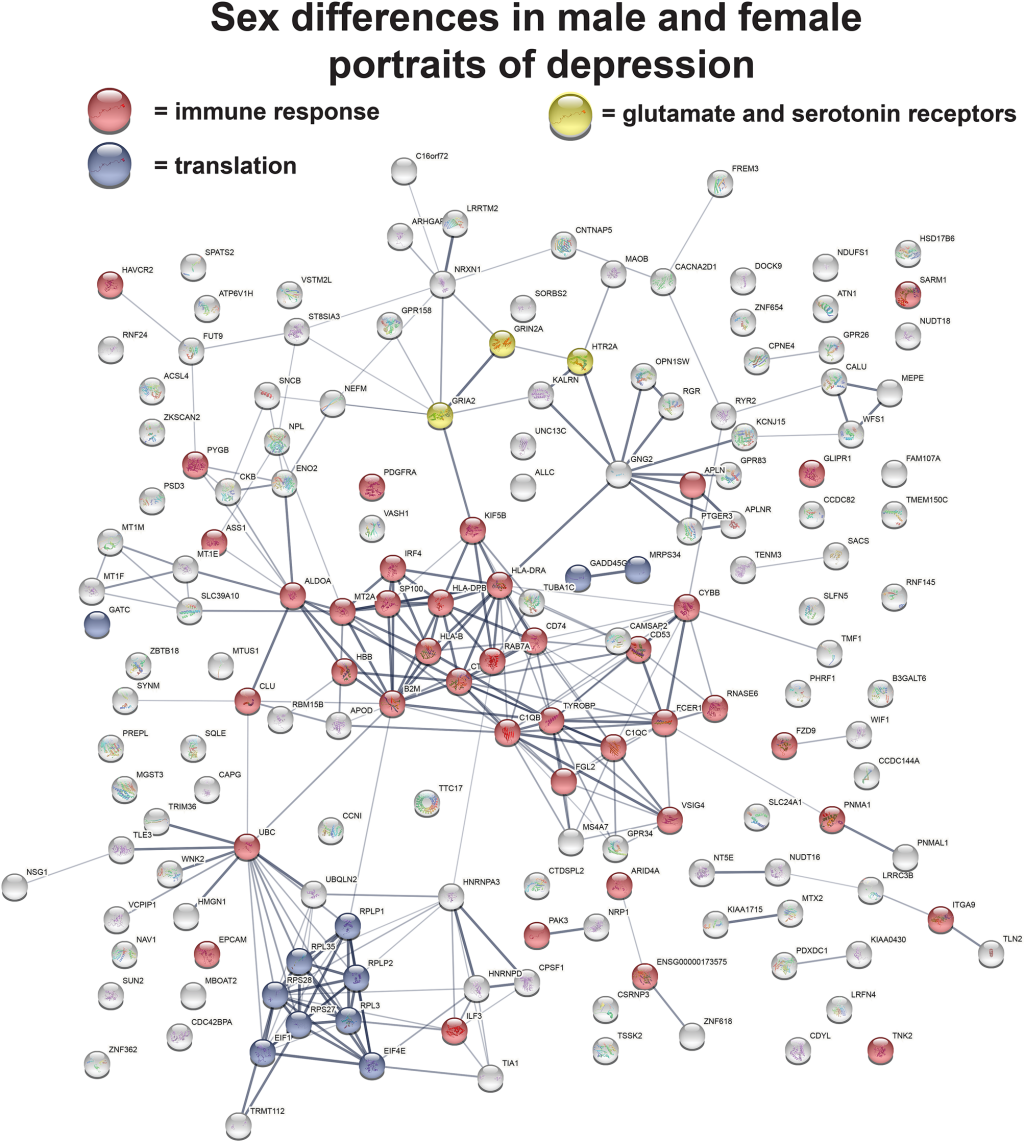
Sex differences in male and female portraits of depression. While overall there was a positive match of the male and female portraits, a subset of genes moved consistently in opposite directions when comparing the top 1000 genes upregulated and top 1000 genes downregulated in both portraits. These opposing direction genes are plotted in STRING (version 11)^18^ that highlights gene–gene interactions. Two areas of enrichment are immune response (red circles) and translation (blue circles). Some neuronal signaling genes of interest include the serotonin receptor, HTR2A, and subunits of the ionotropic glutamate receptor, GRIN2A and GRIA2; all downregulated in males and upregulated in females (yellow circles).

**Figure 3:**
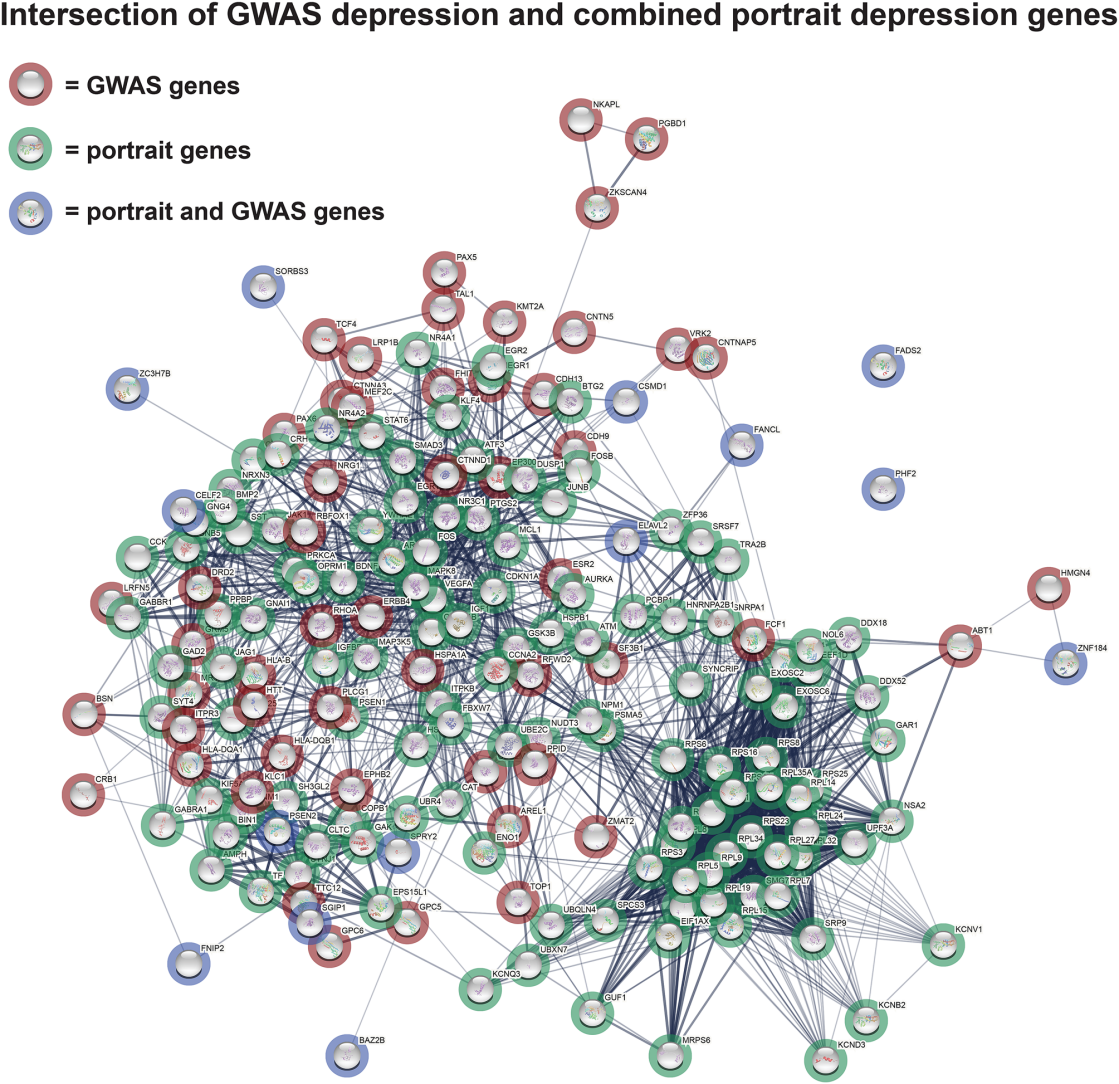
Intersection of GWAS depression and combined portrait depression genes. An interaction of genes from a recent GWAS analysis of depression (red circles) are plotted in STRING (version 11)^18^ with genes from the combined depression portrait (green circles). Genes in common from the GWAS and the top 1000 portrait genes are shown in blue circles. Steps for identifying top interacting genes are provided in the “Methods”. Strength of connection between genes is shown by thickness of lines.

The second approach involved creation of a new combined portrait based on fewer than half of the datasets, but where each was independent from one another in terms of individuals. Then, each of these datasets was randomized to create a new randomly based portrait (see “Methods” for details) and this process was repeated 10 times. The average matching score of the new depression portrait back to its starting datasets was 166.6, while the random average matching was only 130.3 (*p* value < 0.05) (Supplementary Table 1), suggesting that a biological pattern is present in the depression datasets. Finally, when looking within the new depression portrait, the average score for matching downregulated genes (90.4) was higher than for matching upregulated genes (76.2), but it was similar in the random control (65.2 versus 64.6), suggesting a hallmark of depression is a greater consistent downregulation relative to the consistent upregulation of certain genes (Supplementary Table 1).

### Analysis of genetic findings in the portraits of depression

The top dysregulated genes in the combined portrait of depression (Supplementary Table 1) were consistent with a state of low activity or a “hypo” state. For example, the top gene is the downregulation of EGR1, an immediate early gene, but this is not the only immediate early gene with significant downregulation. Others include FOS, FOSL2, FOSB, EGR2, EGR3, EGR4, and JUNB within the top 1000 genes. These genes are reflective of neuronal activity and this collection of downregulated activity genes suggests a state of lowered neuronal activity. Additional genes that reflect cell activity are ARC and the three nuclear receptors from the NR4A family (NR4A1, NR4A2, and NR4A3); these are all downregulated. The idea of “hypo” also extends to some key neuropeptides. For example, downregulation of corticotropin releasing hormone (CRH) is the 6th most dysregulated gene. Other neuropeptides with lower expression in depression are somatostatin (SST) and cholecystokinin (CCK). The glucocorticoid receptor, NR3C1, is also downregulated. One of the enzymes involved in GABA synthesis, GAD2, is significantly downregulated.

Expression patterns of a number of genes suggest a low temperature and/or sleep state with depression. For example, Cold Inducible RNA Binding Protein, CIRBP, is upregulated in the combined portrait and expression of this gene is elevated by lower temperatures and is higher during sleep; it also regulates expression of multiple circadian rhythm genes, including NR1D1. The circadian related gene, NR1D1, is also upregulated. Consistent with a lower CNS temperature, in the top 1000 genes five of five heat shock proteins are downregulated.

Various neurotransmitter receptors were among the top 1000 combined portrait genes, including the GABA B receptor, GABBR1 (downregulated), the GABA A receptor subunit, GABRA1 (downregulated), the purinergic receptor, P2RX7 (upregulated), the serotonin receptor, HTR2C (downregulated), subunits of the ionotropic glutamate receptor, GRIK2 and GRIK5 (both downregulated), and the mu opioid receptor, OPRM1 (upregulated).

The combined depression portrait also includes a number of dysregulated genes (mostly downregulated) involved in the MAP kinase signaling pathway (which has also been strongly implicated in depression, see discussion). Enrichment analysis (e.g., using STRING^18^ or ToppCluster^19^ or Enrichr^20^) of the top 1000 depression genes indicated enrichment for the MAP kinase pathway and genes included five DUSP genes (e.g., DUSP1, DUSP6), four map kinase genes (e.g., MAP2K5, MAPK8), four SPRY genes (e.g., SPRY2, SPRY4), along with SPRED1 and SPRED2 (Fig. 1). Brain derived neurotrophic factor (BDNF), also implicated in depression (see below), is down regulated in the combined portrait (the 164th most dysregulated gene). Given that BDNF and MAP kinase signaling pathways intersect with one another and that both signaling pathways were enriched among the top 1000 dysregulated genes, the interaction of genes in the two pathways was examined using STRING, the protein interaction network. As shown in Fig. 1, genes in the two pathways are highly interactive with one another and a subset are part of the overlap of BDNF and MAP kinase pathways, including BDNF, DUSP1, DUSP4, DUSP5, FOS, NR4A1, VEGFA, and two heatshock proteins. VEGFA is of interest as it is implicated in depression and can use the MAP kinase pathway.

Enrichment analysis also identified general protein kinase activity, regulation of transferase activity, synaptic signaling, neuron development, RNA catabolic process, and viral transcription being among pathways significantly enriched in the top 1000 genes in the combined portrait. Enrichment results from ToppCluster are provided for the top 1000 genes for each portrait in Supplementary Table 1. Using Enrichr and BioPlanet 2019 for enrichment analysis of the top 1000 dysregulated genes, the top five matches were: BDNF signaling pathway (53 genes matching), MAP kinase pathway, cytoplasmic ribosomal proteins, coenzyme A biosynthesis, influenza viral RNA transcription, and translation.

When examining the top 1000 dysregulated genes, just over half, 527, were downregulated in males compared to 594 downregulated genes in females. The male and female portraits have positive matching scores when comparing the top 1000 upregulated and top 1000 downregulated genes in both portraits (see Supplementary Table 1). However, a subset of genes is dysregulated in opposite directions. 87 genes upregulated in males (top 1000 up) are downregulated in females (top 1000 down). Further, 81 genes downregulated in males (top 1000 down) are upregulated in females (top 1000 up) (Supplementary Table 1). Some neuronal signaling genes of interest include the serotonin receptor, HTR2A, and subunits of the ionotropic glutamate receptor, GRIN2A and GRIA2; all downregulated in males and upregulated in females. Enrichment for genes divergent in the sexes was found for both immune response (including C1QB and C1QC) and translation, but otherwise no clear patterns of enrichment were found. Figure 2 provides a plotting in STRING of a subset of genes moving in opposing directions in male and female portraits.

When comparing 268 GWAS genes associated with MDD from a recent study^21^ to the top 1000 portrait genes, 14 common genes were: SPRY2, ELAV2, CELF2, FADS2, FNIP2, ZC3H7B, PSEN2, BAZ2B, SORBS3, FANCL, ZNF184, PHF2, SGIP1, and CSMD1. The hypergeometric overlap of these two lists was not significant. For an additional analysis of GWAS and gene expression results, the top GWAS genes that interacted with one another (n = 53; identified via STRING analysis) and top portrait genes that interacted with one another (n = 123; also identified via STRING) were plotted together in STRING and included the common 14 genes. As shown in Fig. 3, the top GWAS and portrait genes show high interactions with one another, suggesting a possible connection of GWAS to expression outcomes. GWAS genes with high levels of interactions with expression genes were the histone acetyltransferase, EP300, the Rho GTPase, RHOA, the heat shock protein, HSPA1A, the dopamine receptor, DRD2, the glutamate receptor, GRM5, the huntington gene, HTT, and the estrogen receptor, ESR2. Among these highly interactive genes, enrichment included MAP kinase signaling, RNA catabolic process, and neuron development.

### Use of depression portraits to identify potential treatments

**Figure 4:**
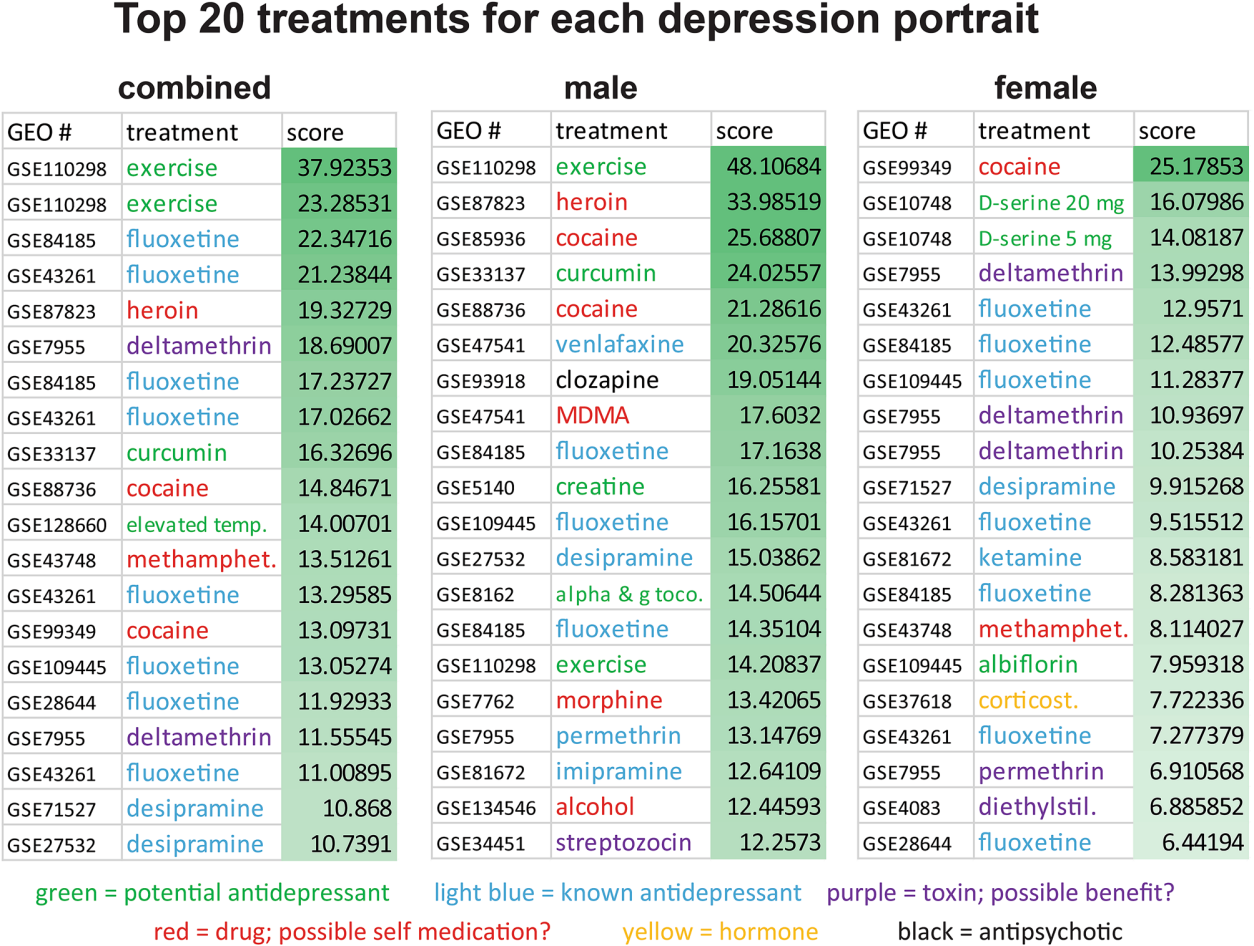
Top overall treatments for each portrait of depression. The top 20 treatments are shown for each portrait. Treatments were placed in one of the following color-coded categories: known antidepressant (light blue); potential new antidepressant (green); drugs of abuse with implications for possible self-medication (red); known toxin, but possible benefit (purple); and hormone (yellow). GEO numbers are provided, but see Supplementary Table 2 for details on comparison made and study. Exercise (data derived from a study in humans) was the top treatment for both the combined and male portraits of depression. Abbreviations are: methamphet. (methamphetamine); a & g tocoph (alpha and gamma tocopherol with vitamin E); diethylstilb. (diethylstilbestrol), corticost. (corticosterone).

The portraits of depression were used with drug repurposing approaches to identify and evaluate potential new treatments for depression (Supplementary Fig. 4). Only datasets were used for analysis that were derived from the nervous system and this step was important because the treatment needs to reverse a brain pattern. The full list of treatments and matches to each of the three portraits is provided in Supplementary Table 2. Figure 4 provides the top 20 matches to each of the portraits. Overall, the top two scoring treatments for the combined portrait were exercise and these treatment data came from human CNS comparing individuals with high versus low or medium lifetime activity (Fig. 4; Supplementary Table 2). The antidepressant, fluoxetine, had eight datasets among the top 20 ranked treatments, including the third overall treatment. Curcumin, the plant chemical from Turmeric, was the ninth ranked treatment (Fig. 4).

When examining known antidepressants, in addition to fluoxetine, other highly ranked antidepressants in order were desipramine, imipramine, venlafaxine, and ketamine. When examining potential novel antidepressants, in addition to exercise and curcumin, some of the top 50 treatments were elevated temperature, D-serine, albiflorin, creatine, alpha and gamma tocopherol, and nicotinamide riboside (Supplementary Table 2). Additional potential treatments were pioglitazone, celastrol, and zinc. A summary of the top 20 potential new treatments for each portrait is provided in Supplementary Fig. 5.

**Figure 5:**
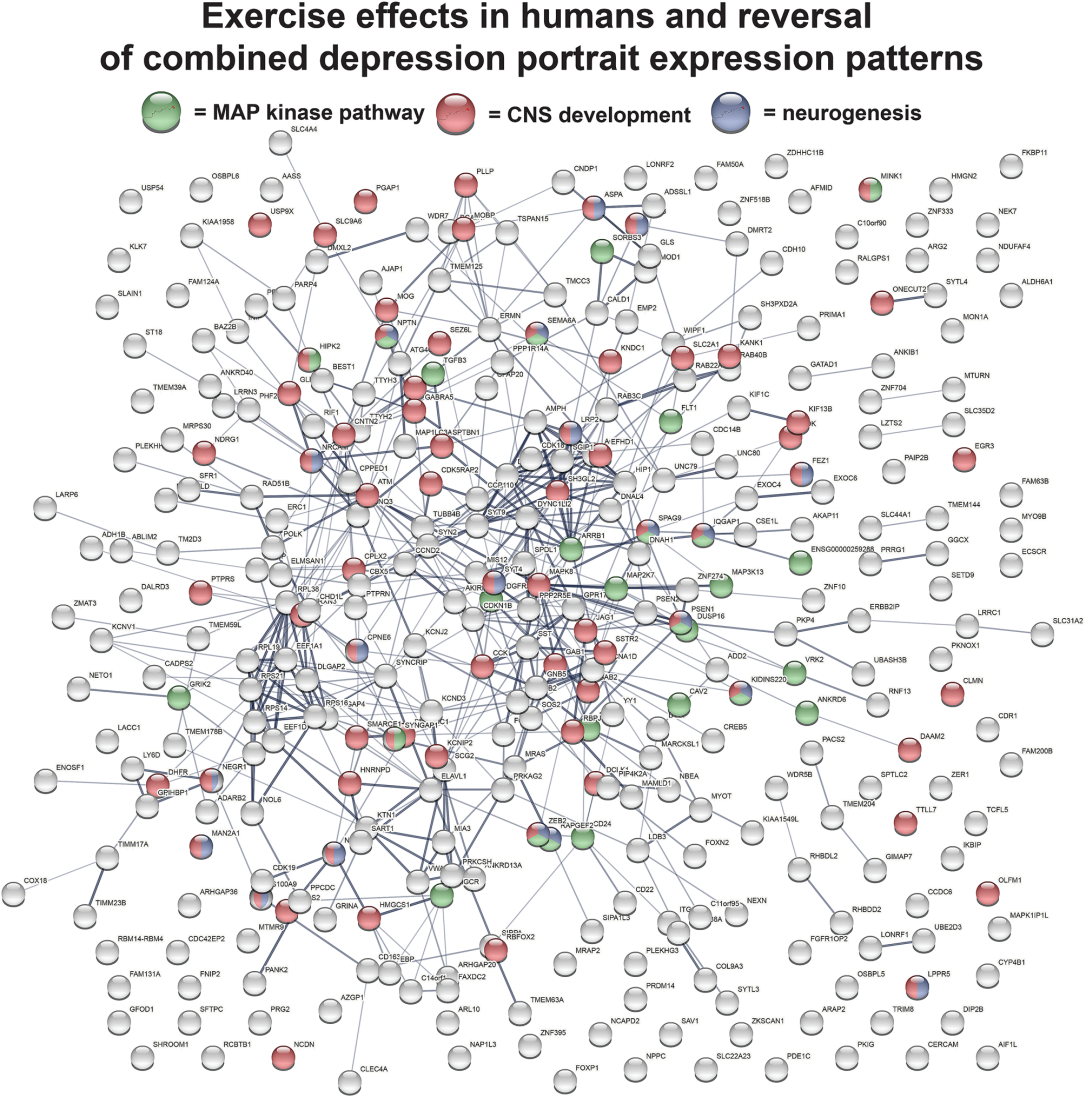
Plotting of genes regulated by exercise that reverse patterns of gene expression in depression. Genes from the top two human datasets that reverse patterns of depression in the combined portrait of depression were plotted in STRING (version 11)^18^ (gene–gene interactions are shown by lines). Enrichment for neurogenesis (blue circles), CNS development (red circles), and MAP kinase signaling (green circles) was found.

For drugs of abuse that could be construed as a form of self-medication, the top scoring drugs included heroin, cocaine, methamphetamine, and morphine and these were among the top 20 treatments (Fig. 4). Among the top 50 treatments were also MDMA, and alcohol. Among known toxins that ranked highly were deltamethrin and chlorpyrifos. Only two hormones were among the top 50 treatments and these were corticosterone and thyroid hormone and neither of these were among the top 30 treatments. The only antianxiety treatment, diazepam, had almost no net effect on altering expression patterns in depression in a given positive or negative direction.

**Figure 6:**
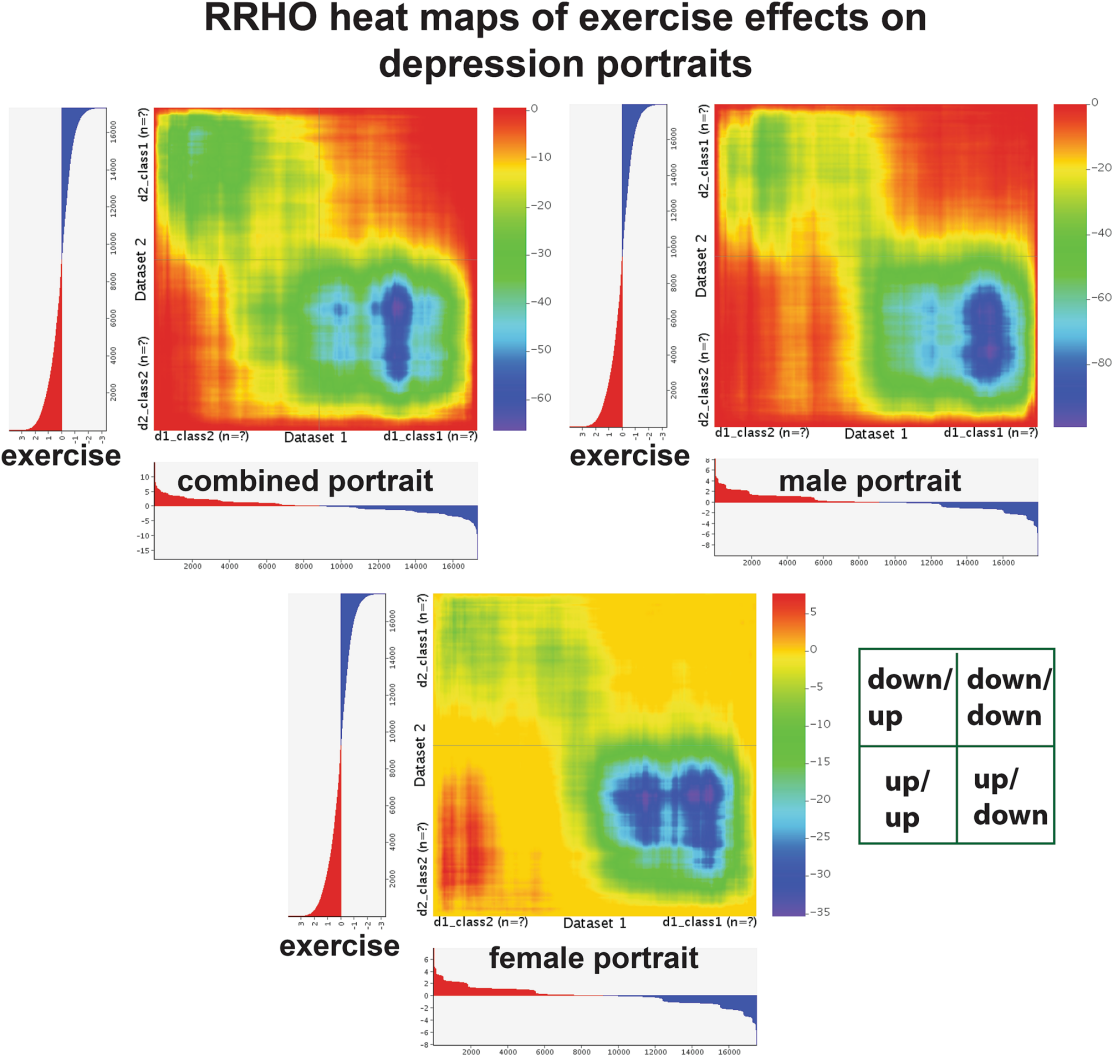
RRHO heat maps of exercise and the portraits of depression. RRHO heat maps^22^ for comparisons of exercise (human dataset) with the three portraits are shown. Y axis is exercise and X axis is for portrait. For each map, the match of direction is shown by the inset. For example, up in exercise and down in depression is the lower right quadrant. Color is –log transformed hypergeometric *p* value showing the strength of the overlap as positive or negative enrichment. In each case there is a high reversal of downregulated depression genes by exercise (blue in lower right quadrant), but in females there are also a subset of genes (red in lower left hand quadrant) that are up in depression and pushed up by exercise. All genes are used in RRHO analysis and profile of upregulated genes are shown in red and downregulated genes shown in blue in the axis for each comparison. Images were made using the web based tool of RRHO^22^. While the current study focusses on the top 1000 upregulated and top 1000 downregulated genes, the mapping here suggests genes outside of this range could be involved in beneficial effects of exercise.

In order to examine more closely how exercise may reverse the combined portrait depression patterns, overlapping genes from the top two human datasets and the combined portrait were plotted in STRING and enrichment for neurogenesis, CNS development, and MAP kinase signaling were found (Fig. 5). Although BDNF was upregulated by exercise, it was not within the cutoff used and is not plotted. As an additional approach, I created a heatmap of how exercise interacted with each portrait using the Rank Rank Hypergoemetric Overlap (RRHO) approach^22^ (Fig. 6). For each portrait, exercise strongly reversed downregulated genes in depression (lower right hand portion of each box) and this extended beyond the top 1000 upregulated and 1000 downregulated genes.

**Figure 7:**
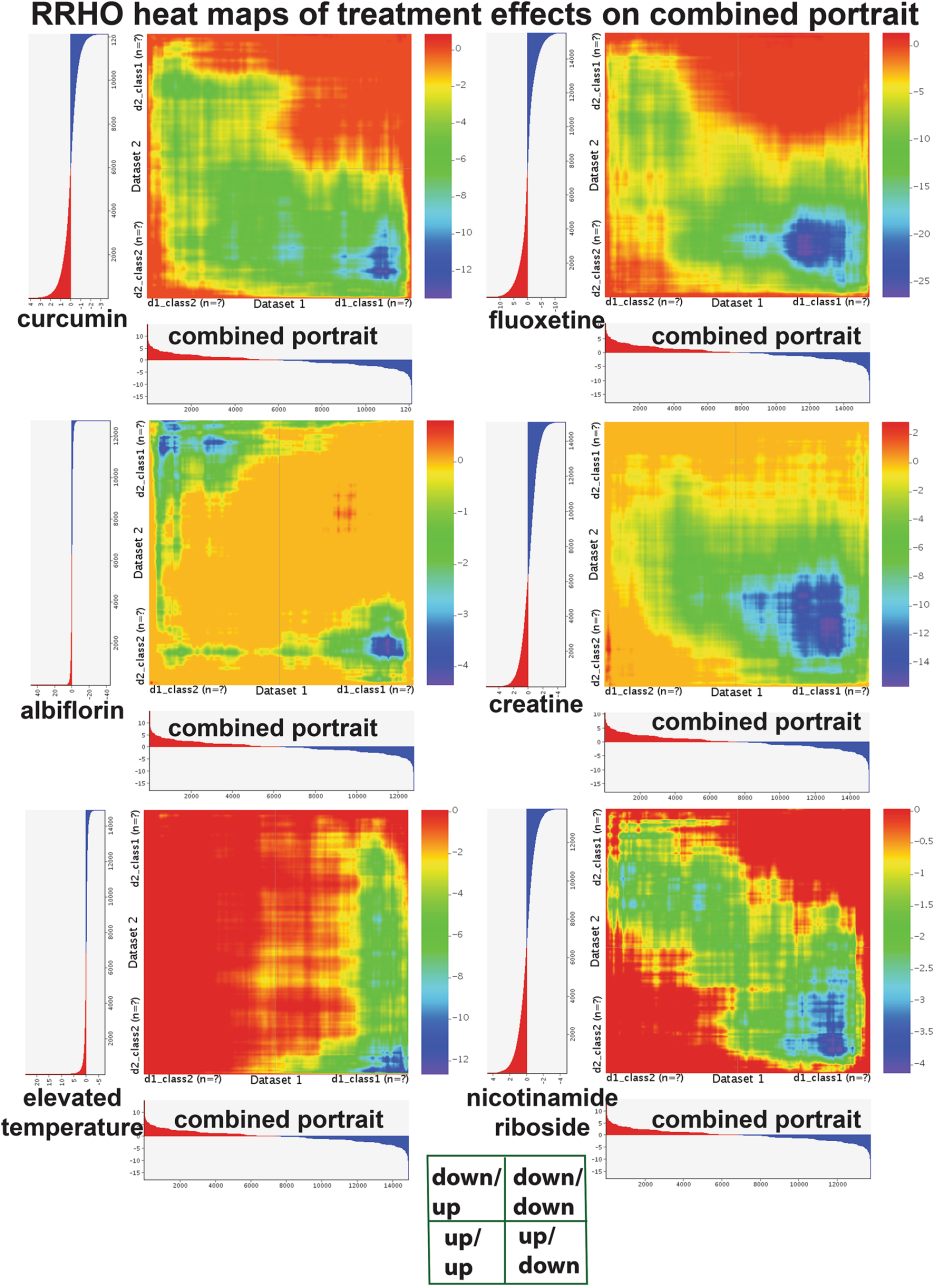
RRHO heat maps of various treatments and the combined portrait of depression. RRHO heat maps^22^ for comparisons of curcumin, fluoxetine, albiflorin, creatine, elevated temperature, and nicotinamide riboside compared to the combined portrait of depression are shown. Y axis is treatment and X axis is for depression portrait. Images were made using the web based tool of RRHO^22^. Details on the axes and colors are same as in Fig. 6. In each case there is a high reversal of downregulated depression genes with the various treatments (blue in lower right hand quadrant), but there are difference in how well each treatment reverses upregulated genes in depression (upper left hand quadrant).

I also created RRHO heat maps for some of the other combined portrait top treatment performers, including curcumin, fluoxetine, albiflorin, creatine, elevated temperature, and nicotinamide riboside (Fig. 7). While each treatment reverses downregulated genes in depression (lower right hand quadrant of each box), albiflorin also shows a signature of reversing upregulated genes in depression. As in the case of exercise, the treatments shown indicate effects that occur outside of the top 1000 up and 1000 downregulated genes.

For the male portrait, the top treatment was exercise (Fig. 4, Supplementary Table 2). Among the top 10 treatments were curcumin, venlafaxine, and fluoxetine. When focusing on known antidepressants, nine of the top 50 datasets were for fluoxetine, but desipramine and imipramine also scored highly and were among the top 50 treatments.

For males, when examining potential novel antidepressants, among the top 50 and in order were exercise, curcumin, creatine, alpha tocopherol with vitamin E, albiflorin, nicotinamide riboside, D-serine, aminolevulinic, and zinc (Supplementary Fig. 5). For drugs of abuse, top scorers were heroin, cocaine, and MDMA (all among the top 10), morphine, alcohol, methamphetamine, methylphenidate, nicotine, and amphetamine (all these among the top 50) (Supplementary Table 2). Three toxins among the top 50 treatments were permethrin, streptozocin, and chlorpyrifos. For hormones, only corticosterone and dexamethasone were among the top 50.

For the female portrait, the top scoring treatment was related to cocaine dependence, but D-serine was the second and third ranked treatment (for two different doses) (Fig. 4, Supplementary Table 2). Fluoxetine was also highly ranked (fifth overall). With a focus on antidepressants, ten of the top 50 treatments were fluoxetine. Ketamine had three entries in the top 50 (the highest at 14th) and this contrasted with the combined portrait and male portrait where none of the top 50 treatments were ketamine. For females, also among the top 50 were desipramine and imipramine.

For females in terms of potential novel antidepressants, among the top 50 were: D-serine (three different doses), albiflorin, exercise, nicotinamide riboside, resveratrol, and curcumin. For drugs of abuse, among the top 50 were cocaine (1st overall), methamphetamine, amphetamine, and MDMA. The toxins deltamethrin (4th overall), permethrin, diethylstilbestrol, chlorpyrifos, bexorotene and bisphenol A were among the top 50. For hormones, corticosterone and thyroid hormone were among the top 50 (Supplementary Table 2).

**Figure 8:**
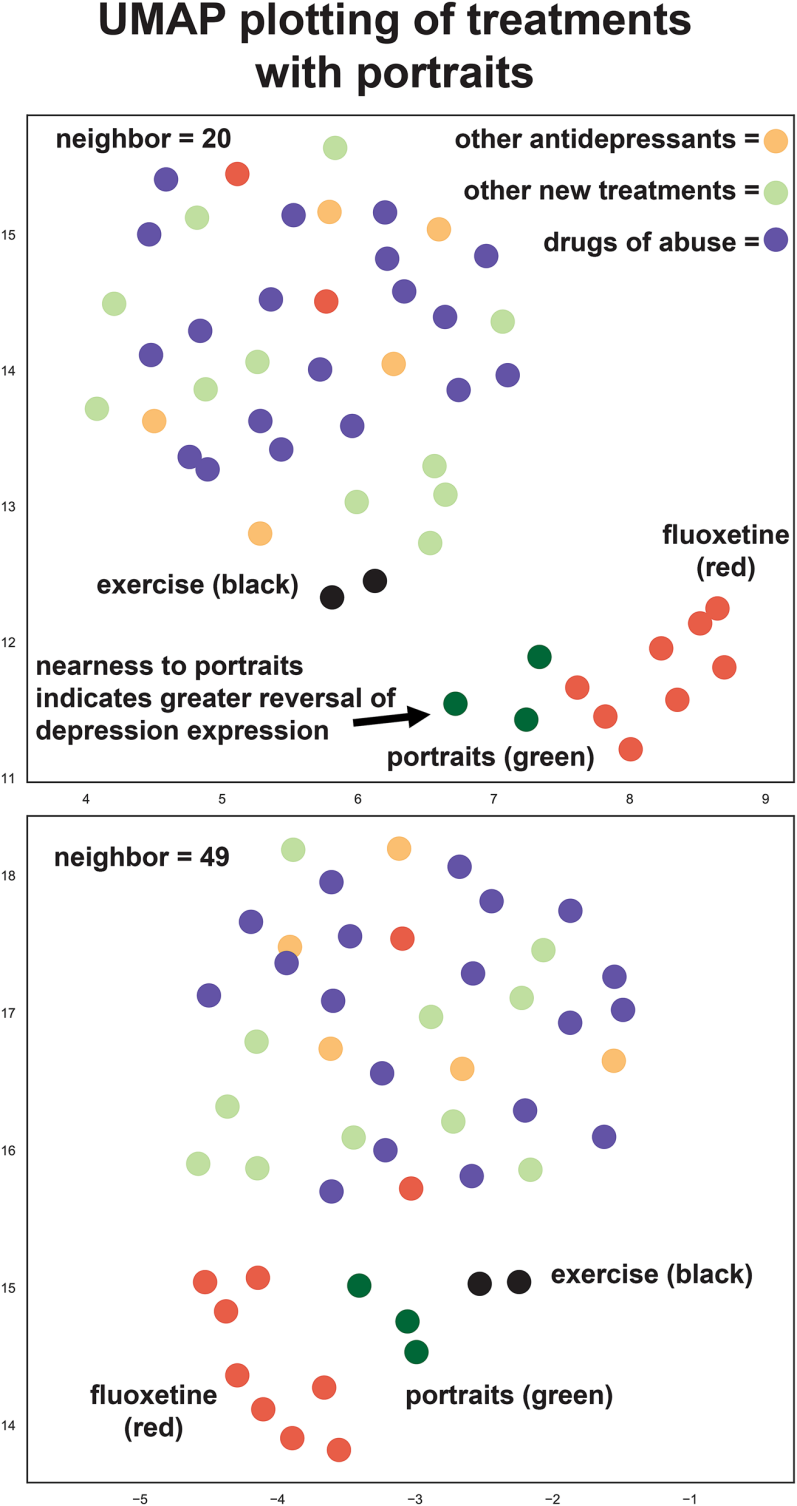
UMAP plotting of treatments with depression portraits. UMAP^23^ was used to incorporate data from complex landscape of multidimensional features (4020 genes) and flatten those to two dimensions to identify similarities between datasets. The sign for the portraits was reversed so that a closer distance (spatial proximity) of a treatment to a portrait represents a better match (reversal of pattern). Plot represents higher dimension data plotted into two dimensions. The x‐ and y‐axes are arbitrary embedding dimensions generated by UMAP. Top graph is from plotting using the neighborhood setting of 20 and lower graph is setting of 49. Overall, the portraits were most consistently matched with fluoxetine and exercise. Colored dots are: green, portraits; red, fluoxetine, brown, exercise (human), purple, drugs of abuse, light green other new treatments, and light yellow, other antidepressants.

Uniform Manifold Approximation and Projection (UMAP)^23^ was used as an alternative approach to gain insights into which treatments may best treat depression as it incorporates the complex landscape of multidimensional features and flattens those to two dimensions. The sign for the portraits were reversed so that if a treatment perfectly reversed all gene expression aspects for a portrait, then it will now perfectly match (be close spatially to) the reversed signed portrait. As shown in Fig. 8, a subset of fluoxetine datasets are close spatially with the three portraits. Among the top novel antidepressants that most consistently matched (were capable of reversing) depression was exercise (in humans).

### Evaluation of an alternative approach for developing a portrait

The current portrait represents one approach and an alternative portrait was created using MetaVolcano. There was significant overlap between MetaVolcano and the present result, but differences likely arose due to differences in approach described in the “Methods” section. The output is provided in Supplementary Table 1. Further, the MetaVolcano portrait was also used to identify potential treatments and those results are presented in Supplementary Table 2. Of interest, the top two treatments were also exercise.

## Discussion

The portrait for depression provides a specific model of a complex gene expression landscape (not just a change in one specific gene) and as a model, it can be tested, challenged, refined, or even replaced. If valid, then the portrait can improve our understanding of what comprises the core of depression and how depression differs in men and women. It also can provide a new metric for evaluating and improving treatments.

One interpretation of the combined portrait of depression is that it is in a “hypo” (i.e., deficient or below normal) state. Numerous immediate early genes that are commonly expressed during neuronal activity were down regulated in association with depression, including the top dysregulated gene, EGR1. Other downregulated immediate early genes include FOS, FOSL2, FOSB, EGR2, EGR3, EGR4, and JUNB. Also consistent with a “hypo” state is the downregulation of the three nuclear receptors from the NR4A family (NR4A1, NR4A2, and NR4A3) that are normally upregulated by activity^24–26^. The activity related gene, ARC^27^, had lower expression as well. This lowered expression of activity-related genes is consistent with the standard, non-medical term depression which is defined as a lower amount or level than usual. Clinically, the Diagnostic and Statistical Manual for Mental Disorders: DSM-5 (DSM) for MDD includes multiple criteria, one being ‘fatigue or loss of energy nearly every day’^28^. It is possible that reduced cellular activity could reflect or trigger this state.

The decreased markers of activity are not the only indicators of a “hypo” state. Among the top downregulated genes are also neuropeptides known to have a connection to depression or mood state. Among these, corticotropin releasing hormone (CRH), the sixth most dysregulated gene, is of interest because higher levels of release are associated with anxiety and arousal and it is known to have a nonlinear effect on behavior when acting among multiple receptors^29–32^. Here, the notable downregulation of CRH suggests a state not necessarily of anxiety, but of low arousal that would be consistent with the DSM criteria of ‘diminished ability to think or concentrate’^28^. Another notable downregulated neuropeptide is somatostatin (SST) that also has been associated strongly with mood and depression^33^. SST often colocalizes with the neurotransmitter, GABA^34^, and one of the enzymes that produces GABA (GAD2) is also downregulated.

Patterns of gene dysregulation consistent with a “hypo” state were found for genes involved in temperature and sleep. The cold inducible gene, CIRBP, is upregulated and most of the dysregulated heat shock proteins are downregulated. Together, these suggest a CNS with a lower than normal temperature and would be consistent with the lowered expression of genes for neuronal activity, such as EGR1 and FOS. CIRBP normally shows daily circadian expression changes with highest levels during sleep and it regulates circadian rhythm genes, including NR1D1^35^, which is also upregulated in the combined portrait. Together, these suggest a potential sleep-like state in depression, which is consistent with the DSM criteria for depression of hypersomnia or insomnia. It should be noted that the elevated CIRBP in the combined portrait is driven more by the male datasets, but in females, NR1D1 is also strongly upregulated and the consistent downregulation of heat shock proteins is also found in females. Consistent with this finding, recent studies have suggested elevation of temperature as a potential anti-depression therapy^36^ and it was of interest that elevated temperature in a lactating model was found to have antidepressant gene expression effects.

The glucocorticoid receptor, NR3C1, was also downregulated and this would support the idea of a lower energy state or low arousal as this receptor is activated in a daily rhythm during the transition from sleeping to waking^37^. Further, lower glucocorticoid receptor expression is consistent with a role for this receptor in the dexamethasone suppression test that can be used to help identify depression^38^.

In terms of neurotransmitter receptors, the GABA B receptor, GABBR1, was downregulated as was a subunit of the GABA A receptor, GABRA1. The glutamate receptor subunits, GRIK2 and GRIK5, the purinergic receptor, P2RX7, and the somatostatin receptor, SSTR2, were also downregulated, but the mu opioid receptor, OPRM1, was upregulated. OPRM1 has previously been linked to depression^39^. Higher OPRM1 against a backdrop of lower neuronal activity could indicate the CNS is poised for, but not getting reward related inputs.

Decreased serotonin signaling has been posited as a contributor to depression^40^ and the serotonin receptor, HTR2C, which has been connected to depression^41^, is downregulated in the combined portrait (680th most dysregulated gene). The drug, lorcaserin, targets this receptor and is used for weight loss and addiction treatments^42^, but treatment effects in depression are not known. No lorcaserin expression datasets were available for analysis, so its potential as potential antidepressant could not be evaluated here. One of the enzymes for producing serotonin, TPH2, is upregulated (1197th most dysregulated), but this does not indicate whether actual release of serotonin is elevated.

The role of BDNF in depression is actively studied^43,44^ and BDNF was the 164th most dysregulated gene in the combined portrait. BDNF is downregulated in depression and the BDNF signaling pathway was significantly enriched in the combined portrait (Fig. 1). Thus, not just BDNF, but related genes are dysregulated in the depression portrait and identification of these associated genes can improve our understanding of how this pathway is disrupted. The BDNF pathway intersects with the MAP kinase signaling pathway as detailed in the KEGG pathways^45^. The MAP kinase pathway is also dysregulated in depression^4,46,47^. MAP kinase signaling was significantly enriched in the combined portrait and Fig. 1 provides a protein interaction map of dysregulated genes that are related to BDNF and/or MAP kinase. This intersection map from the combined portrait provides a more complex picture of these intersecting pathways in depression than past studies.

As shown in Fig. 3, top genes from a recent GWAS survey of MDD^21^ were found among the top dysregulated portrait genes and a number of GWAS and portrait genes interact with one another. Further, for these genes, enrichment for MAP kinase and neuron development was found. Among interesting GWAS genes is SPRY2, which is part of the MAP kinase pathway. Further, PSEN2 is noteworthy as it is linked to Alzheimer’s disease and there is an emerging interest in intersections of depression and dementia^48^. In the depression portrait, PSEN2 is downregulate while PSEN1 is upregulated. Among GWAS genes that interacted the most with depression portrait genes were the histone acetyltransferase, EP300, the Rho GTPase, RHOA, the heat shock protein HSPA1A, the dopamine receptor, DRD2, the glutamate receptor, GRM5, the huntington gene, HTT, and the estrogen receptor, ESR2. Whether or how putative GWAS depression genes interact with or cause gene expression changes in depression is still to be determined.

Out of over 200 depression treatment datasets, exercise was the top rated treatment in the combined and male portrait. The dataset for exercise came from a recent study examining hippocampal gene expression in elderly with known high exercise relative to moderate exercise (top ranked treatment) and versus low activity (second ranked treatment)^49^. RRHO heat map analysis suggested exercise would also advantageously affect genes outside of the top 1000 dysregulated portrait genes (Fig. 6). Using UMAP as an alternative approach to identify the best treatments, exercise again stood out as a promising treatment (Fig. 8). Additionally, exercise ranked highly when examining datasets from rodent models, such as gene expression in the hippocampus in mice with access to a running wheel (33^rd^ ranked top treatment)^50^. A connection between exercise and increased BDNF as part of the antidepressant aspects of exercise have been evaluated^51^. While BDNF is elevated in both of the human exercise datasets, it is not within the cutoff used, so it is not one of the genes matching the portrait datasets. However, for the mouse exercise datasets, BDNF increases are within the cutoff and do match the portraits. The interaction of exercise with depression is complex with apparent contributions of multiple genes in the BDNF and MAP kinase pathways (Fig. 5).

Some of the top ranked non-traditional treatments for depression were of interest because prior work had suggested them as possible antidepressants. For example, D-serine (second highest for females)^52^, curcumin^53^, creatine^54^, elevated temperature^55^, albiflorin^56,57^, alpha and gamma tocopherol with vitamin E^58^, nicotinamide riboside^59^, aminolevulinic acid^60^, celastrol^61^, and zinc^62^ each have been evaluated in some context as a possible treatment for depression. The datasets for D-serine included an unusually high ratio of upregulated relative to downregulated genes overall^63^, though, so additional caution may be needed when interpreting that result.

Hormones that scored well were cortisol and thyroid hormone. Levels of both cortisol^64^ and thyroid hormone^65^ have been linked to depression, so it is not surprising that increases of these hormones in certain contexts would reflect a beneficial treatment effect.

The finding that some drugs of abuse scored highly for reversal of depression patterns is consistent with the idea that in some cases, use of addictive drugs is a form of self-medication^66^. For example, if a drug is altering patterns of gene expression that push patterns in a depressed individual towards normal and this alleviates depression, then it is possible the addictive drug would have both the typical reward but also reward in the form of alleviation of symptoms. For the female depression portrait, cocaine dependence^67^ was the top treatment. Also, when examining the genes affected by cocaine, many were related to cellular activity. Drugs of abuse are not suggested as a possible treatment for depression, but an understanding of how they might interact with depression opens the possibility of targeting specific pathways in developing new treatments. Similarly, some of the treatments with possible antidepressant actions, such as deltamethrin, are known to have toxic effects^68^. Similar to drugs of abuse, it is not suggested a potential toxin be taken as a treatment, but by examining how these types of chemicals could potentially reverse aspects of depression could open new lines of treatments.

The scoring system used allows one to identify possible treatments for depression that could make things worse, namely by pushing expression patterns in the same direction as in depression. For antipsychotics, while some ranked among the top 50 for treatments, many were found to have adverse effects. For example, for the combined portrait, haloperidol and clozapine had seven entries for these drugs in the bottom 50 treatments (most adverse effect on expression), including the worst (clozapine) and second worst (haloperidol) treatments. Interestingly, the one clozapine study showing a positive antidepressant effect was performed on a HDAC2 knockout in forebrain neurons mouse line^69^, suggesting potential beneficial effects of this antipsychotic may be dependent on the genotype.

Fluoxetine was the best performing traditional antidepressant, but multiple others, including desipramine and imipramine also scored well. Ketamine treatment had mostly positive effect in females, but not in males or the combined portrait. A limitation for the ketamine datasets was they were based on a single injection approach^70^. No studies available examined long term effects of ketamine treatment and thus comparisons to other studies on antidepressants using chronic long term treatment are not available.

The depression portraits for males and females had large consistent overlaps of genes in the same direction, but had a subset of genes that are significantly dysregulated in opposite directions. Among notable receptors, the serotonin receptor, HTR2A, and the glutamate receptor, GRIN2A, were both upregulated in females and downregulated in males. Enrichment of genes for translation and for the immune system (e.g., C1QB and C1QC) were identified (Fig. 2). The sex differences in C1QB and C1QC are of interest as they have been implicated as central hubs of gene expression in the hippocampus with depression^71^. Otherwise, no additional clear enrichment pattern was found for genes dysregulated in males and females with depression in opposite directions. The finding of sex differences in depression is consistent with previous work^4^.

One limitation for evaluating sex differences in response to treatments is that the majority of the treatment datasets were performed in males and this could make treatments appear to work better in males. Given this major and possibly explanatory caveat, a general finding was that when comparing the top most effective treatments between males and females, the magnitude of reversal (overall score) was lower in females suggesting that the specific gene landscape in depression is harder to treat in women. Differences in response to treatments have been observed between men and women with depression^72,73^. However, without more treatment datasets balanced equally between the sexes, it is hard to substantiate sex differences here in terms of response to treatments.

There was a high concordance between the present approach and MetaVolcano results. For example, all activity related genes were found in both approaches in the top 1000 genes. Differences likely reflected differences in approach as detailed in the “Methods” section. The use of multiple approaches to establish a portrait is valuable and a consensus on how to best create a portrait will likely only come with future studies and analysis.

The current study provides a portrait of depression and holds the promise of providing a more complete understanding of the basis of depression while also providing a tool for identifying and developing new treatment approaches. However, the portrait of depression has limitations and the following is a list of limitations and future directions intertwined.

1. While the approach used highlights consistent gene expression patterns in depression, it is difficult to untangle the cause from effect and likely there is an interaction of both. For example, dysregulation of some genes may be causative, some may be in response to depression, and others may be working to oppose those effects and thus be neuroprotective. Further, the underlying datasets include data from individuals with different possible life histories including exposure to antidepressants and/or drugs of abuse that in turn can affect gene expression. This concerns is mitigated somewhat by the approach of highlighting consistent changes with depression and discounting inconsistent changes that may occur to different life history events. In the future, it will be valuable to try to evaluate which gene patterns are the most causative, which may be a response to depression, and which (if any) may seem like a dysregulation but are neuroprotective.
2. The combined portrait is likely to be the most robust as it incorporates data from males, females, and mixed cohorts. The male and female portraits are valuable, but because they will always be built from less data, it is worth adding extra caution here and in future studies. Further, multiple cutoff decision points were used in creating the model and it is possible there are better ways for weighting genes. While the addition of future human datasets will improve the model, we should also work to improve and evaluate multiple approaches, including MetaVolcano, for developing the portrait. Additionally, the portraits were built from different brain regions that include a mixture of neurons and glia and this approach can be useful for identifying global consistent dysregulation in depression. However, with the emergence of high throughput single cell RNA-seq, complementary approaches of global versus single cell RNA changes will be important in refining an understanding of depression. Further, different forms of depression occur, including typical and atypical, and while the current study does not distinguish among these, in future studies it would be beneficial to develop different portraits for different versions of depression.
3. Caution should be used for evaluation of possible treatments given that the expression studies varied widely across multiple factors, including sex, species, numbers, brain region, treatment length (some were acute, some were chronic for months), and platform (microarray or RNA-seq). Thus, for use of current and future treatment datasets, an understanding of experimental design is relevant for interpretation.
4. A potentially useful step could involve use of the depression portraits to evaluate and identify what animal model has the highest concordance with the depression brain signature. This approach includes comparing the portraits for depression with gene expression patterns from various animal models (e.g., transgenics, stress exposure). While there is some debate about whether there is or ever can be a good animal model for depression^74,75^, identification of a model that closely matches the portraits for depression would be of use. In only a few of the 200+ treatments was the treatment analyzed in the context of a model that included depressive-like characteristics. With the right model, in future studies one could evaluate treatments that reverse a preexisting dysregulated expression pattern that matches depression in humans.
5. A goal of producing a portrait for depression is to gain new insights into depression but also to produce a platform for evaluating and identifying promising new treatments. Here, antidepressants performed well, but there is ongoing concern about treatments that themselves create issues, including adverse effects with withdrawal as can occur with venlafaxine^76^. A potentially promising approach is to identify multiple complementary treatments whereby each treatment itself has a positive impact and risk of withdrawal effects are low. By examining large-scale gene expression alterations with treatments and combinations of treatments against the depression portrait, it is possible there is a path forward for understanding and identifying new treatments for depression.

## Methods

### Creation of gene expression portraits of depression

An overview of the “Methods” is provided in Supplementary Fig. 1. I began by identifying publicly available large-scale gene expression datasets from the CNS of depressed and control individuals within Gene Expression Omnibus and if possible, I used the GEO2R tool to produce a differential gene expression (DGE) for each study^77^. Supplementary Table 1 provides a list of all datasets used. For GSE102556 the datatype (RNA-seq) could not be analyzed by the GEO2R tool, the dataset was not available for analysis in the GEO RNA-seq Experiments Interactive Navigator (GREIN)^78^, and the original publication did not include a full DGE listing. Therefore, I ran analysis on the raw data using EdgeR^79^ to produce a DGE list for each sex and brain region. Top differential genes were similar, but not identical to the original publication^4^ likely due to differences in the settings used within EdgeR. A DGE list provides a list of genes sorted by *p* value from most to least significantly dysregulated for a given study. It also provides information on the direction of change. For this study, a positive sign indicates that a gene is upregulated in depression, whereas a negative sign indicates downregulation. As a next step, the *p* value for each gene was − log10 transformed (e.g., a *p* value of 0.000001 becomes 6), so that lower *p* values become larger positive numbers. This number was then multiplied by the sign of the direction of change. Therefore, a significantly upregulated gene may be noted by a 6, while a downregulated gene may be noted by a − 6. Each expression study was converted to a single file with two columns, one for Gene.symbol and the other called sign1, which provides the information on significance and direction of change. This approach is used because it is the standard for preparing data for conducting rank rank hypergeometric analysis^22^. As a next step, I updated the HUGO gene symbols for each dataset. This is necessary because the datasets were produced at different time points and the annotations for human genes have been regularly changing. I ran into the issue that older datasets with certain problematic older symbols, such as MARCH3 (now called MARCHF3), are turned into calendar dates if the data are opened in Excel^80^. To perform this gene symbol update, I created a list of older gene symbols along with the corresponding updated symbol and included script to catch any symbols that may have been inadvertently turned into a date. I also identified a subset of gene symbols that are difficult for updating because at some point a currently valid symbol was turned into another currently valid symbol. To address this issue I avoided updating any currently valid gene symbol. The script in R and files for updating the gene lists used in this study are available upon request.

For the creation of each portrait, I used the datasets indicated in Supplementary Fig. 1 and Supplementary Table 1. A few of the datasets were excluded and the rationale was to mitigate the effect of overrepresentation of the same individuals and to exclude a dataset that included only about half of the normal number of genes; see Supplementary Table 1 for details. The following number of datasets was used: combined portrait (29 datasets), male portrait (13 datasets), and female portrait (13 datasets). After formatting each dataset as describe above, I then combined all relevant datasets based on gene symbol. As part of this process I began by ranking genes within each dataset based on upregulated genes and ranked them from low to high (e.g., 1 = most significantly upregulated gene; the largest number = the most significantly downregulated gene). I removed any gene if more than a third of the datasets had missing values. For any remaining missing values I entered the mean of the final gene count. The rationale is that with the mean rank, these values will be ignored when evaluating the top up and downregulated genes. Using a cutoff at 1000 increments, I began by examining each gene and counting how many datasets it received a count of 1000 or lower. I then used the same approach to count 2000 or lower and continued in this manner up to 8000 or lower. I then focused on downregulated genes so that now the ranking is based on downregulated genes (e.g., 1 = most significantly downregulated gene). I used the same format of counting as for upregulated genes. For example, I counted how many times a given gene was in the top 1000 downregulated genes, the top 2000 genes, up until 8000.

The idea of the portrait is to identify the top dysregulated genes in depression based on significance and consistency of direction of expression change in a certain direction. Given this, I created a scoring system for each gene using the data from above in multiple stages. First, I took the score of the number of datasets in which a particular gene was in the top 1000 for upregulation. For example, for the combined portrait for the gene, RPS23, this was 13 datasets so the score was 13. I then subtract the number of times the gene occurred in the top 1000 downregulated genes. For RPS23 this was zero. By subtracting the downregulated number from the upregulated number, the starting score for RPS23 in the combined portrait was 13.0. For EGR1, though, the number was 0 for top 1000 upregulation (i.e., there were no cases where EGR1 was in the top 1000 upregulated genes) and 16 for top 1000 downregulation, so the starting score was − 16.0. The idea is that the genes that rank highest (most positive) and lowest (most negative) are those that are consistently different in a certain direction. If a gene is found in the top 1000 upregulated genes in 5 lists, but downregulated in the top 1000 in 5 lists, then these will cancel out and its starting score would be zero. In order to expand the analysis to the top 2000 up or downregulated genes, I counted how many times a gene met that criterion, but then multiplied it by 0.1, so that its impact was tenfold less than for the top 1000 genes. For the top 3000, I used the same approach, but now multiplied by 0.01, so it was reduced by a factor of 10 again. I continued in this approach up until the top 8000 up or downregulated genes. I then added all the numbers for a given gene. For RPS23, the final number was 14.76545 and for EGR1 it was − 17.8769231. Thus, the numbers in the portrait provide information back into how it was represented among the starting datasets. As a final step I sorted the genes by the absolute value so that the final list contains all genes ordered by magnitude of the difference from controls and then added information on direction of expression change (up = positive sign, down = negative sign). I provide script that runs in R and all the starting datasets needed to replicate creation of the portraits are both publicly available and available upon request. The output in this format allows for portraits to be compared with the original datasets, with one another, and with treatments (see below); also the format can be easily used for RRHO analysis^22^. In Supplementary Table 1, I provide an output from R just prior to creation of the final combined portrait so the reader can examine any gene in detail. Using this approach, I created three portraits for depression; one that included both men and women (combined portrait), one for males only (male portrait), and one for females only (female portrait). The complete portraits, plus lists of the top 1000 upregulated genes, the top 1000 downregulated genes, and the top 1000 overall dysregulated genes are provided for each portrait in Supplementary Table 1.

An alternative approach to create a depression portrait was also explored using the newly developed tool, MetaVolcano^11^. The output from MetaVolcano is provided in Supplementary Table 1 along with a comparison to the current findings. Key differences in approach are that MetaVolcano includes a focus on specific *p* values and fold change levels, whereas the present study uses *p* value for ranking and fold change for direction and each round of analysis involves the same number of genes from each study. Thus, a difference is that MetaVolcano may emphasize genes with particularly low *p* values or high fold change from a subset of studies whereas the current approach uses a ranking system so each dataset contributes equally to the final model. Further, MetaVolcano maintains all genes in final output whereas this study only evaluates genes present in more than two thirds of the samples.

### Testing individual depression datasets against the three portraits and one another

Each original depression dataset was evaluated with the three portraits using a rank rank hypergeometric approach comparing the top 1000 upregulated and top 1000 downregulated genes from each dataset. From these comparisons I determined the *p* value and then used the − log10 (*p* value) transformation so that lower *p* values are represented by higher positive numbers. I used the labeling system as used in the rank-rank hypergeometric publications of: A, upregulated in both groups; B, up in first group, but down in second; C, down in first group, but up in second; D, downregulated in both groups^22^. I then created a score where the output of A (up in both) and D (down in both) were added together and then the outputs of B (opposite directions) and C (opposite directions) were subtracted to provide a final score. Portraits were compared against one another using the same approaches as above to provide insights into how well the male and female portraits matched the combined portrait and to identify sex differences. The outputs of all comparisons with the individual depression datasets with the three portraits are provided in Supplementary Figs. 2 and 3 and Supplementary Table 1. I also provide a script that runs in R for conducting the comparisons.

### Control portrait

In order to evaluate whether the portraits were capturing a consistent biological signature, I selected 12 depression datasets (5 male, 5 female, and two combined sexes) and created a new depression portrait based on reduced input. Importantly, none of these were from the same individuals so patterns, if any, should emerge from a biological signature. Then, within each dataset, I randomized how the genes and data were associated and created a control random portrait based on the randomized data. I then compared each of the respective starting components against the respective portraits using approaches above to see how well the model fit. The creation and analysis of the random portrait was repeated 10 times and the results averaged. A one-way ANOVA was performed on the overall scores between the new depression portrait and random portrait. Data used and results are provided in Supplementary Table 1.

### Analysis of the top dysregulated genes

Enrichment analysis for significance (*p* < 0.05) was conducted using ToppCluster^19^, Enrichr^20^, and STRING^18^ on the top 1000 dysregulated genes in the three portraits. I used the cutoff of 1000 because this is expected to reflect a biological signature and multiple MDD expression datasets have over 1000 significantly dysregulated genes. The reader is encouraged to explore results using different cutoffs. The top 1000 gene lists are provided in Supplementary Table 1 and can be entered into enrichment tools. Results from some of the enrichment tests, including from ToppCluster, are provided in Supplementary Table 1. I also entered subsets of genes that were enriched in certain pathways (e.g., MAP kinase and BDNF signaling) into STRING to gain insights into protein interaction networks^18^.

### Analysis of GWAS depression genes with the combined portrait of depression

268 genes from a recent GWAS analysis of depression^21^ were compared with the top 1000 dysregulated genes from the combined portrait to identify common overlapping genes (N = 14) and lists were compared using a hypergeometric test. Further, the 268 GWAS genes were analyzed in STRING and the genes with the highest connections with one another (at least 4 gene–gene interactions) were identified (N = 55). The same approach was used for the top 1000 portrait genes to identify the top interacting genes (at least 19 gene–gene interactions) (N = 124). These two sets of genes along with overlapping genes were entered together into STRING to evaluate interactions and perform enrichment analysis.

### Collation and preparation of potential treatment datasets

An overview of the approach used is provided in Supplementary Fig. 4. In order to identify potential treatments for depression I used two drug repurposing tools, NIH LINCS L1000^81^ and Enrichr^20^. The drug repurposing approach of sign matching is based on the premise that if one knows the direction of change of gene expression in a disorder and one knows the direction of gene expression change produced by a treatment, then one can identify treatments that move expression of the genes in the disorder back to the neurotypical state. In L1000 I entered 1000 genes upregulated and downregulated for each portrait and used the reverse feature to identify drugs that could reverse the gene pattern. For Enrichr, I used the Crowd feature and entered the upregulated and downregulate genes in separate sessions and retrieved results from the appropriate Drug Perturbations from GEO output. For example, for an input of upregulated depression genes, I extracted results from GEO down. For both tools, many of the datasets used are not neural tissue (e.g., a breast cancer cell line). I used the results from both approaches to create a starting list of potential treatments, but then went to GEO datasets^77^ and searched for and analyzed only studies that had been performed using CNS or related tissue (e.g., neuronal stem cells) so that the treatment would have relevance to CNS dysregulation in depression. Only datasets with a minimum of three per group were used. Not all potential treatments (for example, imatinib) had datasets from the CNS, so these could not be explored further. The two drug repurposing tools had some limitations as they did not include certain types of datasets such as exercise (or wheel running in rodents) effects on expression changes. I added to my search any additional treatment that had some evidence for a potential therapeutic effect on depression (such as exercise). Many of these studies were not specifically looking at potential treatments for depression, but were examining an experimental treatment in a different context. A majority of the datasets were from mice and rats, but other datasets were from humans, non-human primates, and neuronal tissue, including neuronal cell lines. A full list of all treatments tested is shown in Supplemental Table 2. When possible, I used the GEO2R tool (designed to work with microarray data) to obtain a differential gene expression output. RNA-seq analysis was in some cases performed using GREIN if the datasets were available^78^. Together, I processed over 200 datasets. As a final step, I used the same approach of transforming the *p* value via the –log10 approach and multiplied this by the sign of direction of change so the formatting is identical to that for datasets used in the creation of the portraits. The treatment datasets are publicly available and versions that are formatted for use with the software tools provided are available upon request.

### Use of portrait to identify potential treatments

I tested each potential treatment dataset separately against the three portraits using a rank rank hypergeometric approach comparing the top 1000 upregulated and top 1000 downregulated genes from each dataset. This approach was similar to the one used above for evaluation of individual depression datasets to the portraits. Here, though, I created a final score where the potential therapeutic effects were first summed: outputs of B (treatment reversed genes down in depression) and C (treatment reversed genes up in depression). I then subtracted effects where treatment could be viewed as worsening the state of depression, namely output of A (treatment upregulates genes already up in depression) and D (treatment downregulates genes down in depression), to get a final score with a higher positive number reflecting higher potential reversal of depression profiles. Thus, a treatment that reversed a high number of depression portrait genes, but had little effect on pushing genes in the same direction would receive a high score. The outputs of all comparisons with the individual depression datasets with the three portraits are provided in Supplementary Table 2. I provide a script that runs in R for conducting the comparisons. I also provide script in R that allows one to easily compare a given treatment with a given portrait, to identify the specific genes modified by the treatment that are dysregulated in depression. The focus on the top 1000 upregulated and top 1000 downregulated genes across treatments allowed for a uniform comparison between treatments and is consistent with the approach by Enrichr that examines 500 genes. I explored using RRHO^22^ for treatment to treatment comparisons, but this can provide a disparity in cutoffs. For example, for one treatment RRHO may examine 5000 up-up genes and 100 up-down genes, but for a different treatment vastly different cutoffs may be used. Thus, comparison among over 200 treatments is difficult and the planned set cutoffs were used. Further, RRHO is designed for use with two gene expression datasets and while the portraits are formatted in a manner consistent with a gene expression study, they are not expression results per se. However, the data are formatted to work with RRHO and script for use in R is provided to produce an output between any treatment and any portrait that then can be entered into RRHO for analysis. I used the heatmap output from RRHO to highlight the reversing of depression expression by some of the treatments with portraits and this is also useful when examining patterns outside of the top 1000 up or 1000 downregulated genes.

### Uniform Manifold Approximation and Projection (UMAP) analysis

UMAP^23^ was used as alternative exploratory approach to gain insights into the performance of some of the top treatments. UMAP incorporates data from complex landscape of multidimensional features (e.g. genes), flattens those to two dimensions to identify similarities or differences between datasets. I used some of the top treatments and the portraits, but I reversed the sign of the portraits so that if a theoretical treatment perfectly reversed all gene expression aspects for a portrait, it will now perfectly match (or be closely aligned with spatially) that portrait. Hence, the closer treatments are spatially to portraits, the better the reversal and potentially better treatment. For the analysis shown, the neighborhood size was set for two different sizes and the correlation clustering tool was used. Because UMAP works well with higher dimensions, 4020 genes were used and these were derived from the top 1000 up and 1000 down regulated genes from the three portraits.

## Supplementary Information

Supplementary Information 1.

Supplementary Figures.

Supplementary Table 1.

Supplementary Table 2.

## Data Availability

All original datasets are publicly available datasets as indicated. Datasets were transformed and reformatted as indicated and those versions that were used in this study are available upon request. Script is provided to allow for replication of results and modification of script. Output files are also provided in Supplementary Information. Any additional information can be received from the author by request.
