# Supplementary material for: Creation of a gene expression portrait of depression and its application for identifying potential treatments
Source: Sci Rep. 2021 Feb 15;11:3829. doi: 10.1038/s41598-021-83348-0 (PMC7884719; doi:10.1038/s41598-021-83348-0)
Supplement: Supplementary file 1 — Supplementary Information 1. [file 41598_2021_83348_MOESM1_ESM.pdf]

This text will create a combined depression portrait (z1) that includes males and females. The output "FINAL.txt" is the same as z1, but has a different name so results can be compared with z1. All input files are from publicly available datasets and the files referred to here are available on request. Note that the file HUGO2.txt is simply a list of all HUGO gene symbols that allows for the joining of datasets.

Required packages: plyr and dplyr

```
h1 <- read.table ("x1.txt", header = TRUE)
h2 <- read.table ("x2.txt", header = TRUE)
h3 <- read.table ("x5.txt", header = TRUE)
h4 <- read.table ("x6.txt", header = TRUE)
h5 <- read.table ("x7.txt", header = TRUE)
h6 <- read.table ("x8.txt", header = TRUE)
h7 <- read.table ("x9.txt", header = TRUE)
h8 <- read.table ("x10.txt", header = TRUE)
h9 <- read.table ("x11.txt", header = TRUE)
h10 <- read.table ("x12.txt", header = TRUE)
h11 <- read.table ("x13.txt", header = TRUE)
h12 <- read.table ("x14.txt", header = TRUE)
h13 <- read.table ("x15.txt", header = TRUE)
h14 <- read.table ("y1.txt", header = TRUE)
h15 <- read.table ("y2.txt", header = TRUE)
h16 <- read.table ("y5.txt", header = TRUE)
h17 <- read.table ("y6.txt", header = TRUE)
h18 <- read.table ("y7.txt", header = TRUE)
h19 <- read.table ("y8.txt", header = TRUE)
h20 <- read.table ("y9.txt", header = TRUE)
h21 <- read.table ("y11.txt", header = TRUE)
h22 <- read.table ("y12.txt", header = TRUE)
h23 <- read.table ("y13.txt", header = TRUE)
h24 <- read.table ("y14.txt", header = TRUE)
h25 <- read.table ("y15.txt", header = TRUE)
h26 <- read.table ("y16.txt", header = TRUE)
h27 <- read.table ("xy1.txt", header = TRUE)
h28 <- read.table ("xy2.txt", header = TRUE)
h29 <- read.table ("xy4.txt", header = TRUE)

newlist <- list(h1, h2, h3, h4, h5, h6, h7, h8, h9, h10, h11, h12, h13, h14, h15, h16, h17, h18, h19, h20, h21, h22, h23, h24, h25, h26, h27, h28, h29)
hhh <- read.table ("HUGO2.txt", header = TRUE)

ranksup <- function (x) {
x$uprnk <-  rank(-x$sign1, ties.method ="average")
rr1 <- x[ , c (1,3)]
zzz6 <- join(hhh, rr1, by="Gene.symbol")}

gdone <- lapply(newlist, ranksup)

tt <- as.data.frame (gdone)
names (tt) [names (tt) == 'Gene.symbol'] <- 'temp1'
tt3 <- select(tt,-starts_with("Gene"))
names (tt3) [names (tt3) == 'temp1'] <- 'Gene.symbol'

tt3$nacount <- apply(is.na(tt3), 1, sum)

colinfo <- (ncol (tt3)) -2
colinfo1 <- ceiling (colinfo * (1/3))
n1 <- colinfo +3
n2 <- n1 + colinfo -1

yy3 <- tt3 [which (tt3$nacount <= colinfo1) , ]
yy3[is.na(yy3)] <- nrow(yy3)/2

yy3$rnk1  <-  rank(yy3$uprnk, ties.method ="average")
yy3$rnk2 <-  rank(yy3$uprnk.1, ties.method ="average")
yy3$rnk3 <-  rank(yy3$uprnk.2, ties.method ="average")
yy3$rnk4 <-  rank(yy3$uprnk.3, ties.method ="average")
yy3$rnk5 <-  rank(yy3$uprnk.4, ties.method ="average")
yy3$rnk6 <-  rank(yy3$uprnk.5, ties.method ="average")
yy3$rnk7 <-  rank(yy3$uprnk.6, ties.method ="average")
yy3$rnk8 <-  rank(yy3$uprnk.7, ties.method ="average")
```

```
yy3$rnk9 <-  rank(yy3$uprnk.8, ties.method ="average")
yy3$rnk10 <-  rank(yy3$uprnk.9, ties.method ="average")
yy3$rnk11 <-  rank(yy3$uprnk.10, ties.method ="average")
yy3$rnk12 <-  rank(yy3$uprnk.11, ties.method ="average")
yy3$rnk13 <-  rank(yy3$uprnk.12, ties.method ="average")
yy3$rnk14 <-  rank(yy3$uprnk.13, ties.method ="average")
yy3$rnk15 <-  rank(yy3$uprnk.14, ties.method ="average")
yy3$rnk16 <-  rank(yy3$uprnk.15, ties.method ="average")
yy3$rnk17 <-  rank(yy3$uprnk.16, ties.method ="average")
yy3$rnk18 <-  rank(yy3$uprnk.17, ties.method ="average")
yy3$rnk19 <-  rank(yy3$uprnk.18, ties.method ="average")
yy3$rnk20 <-  rank(yy3$uprnk.19, ties.method ="average")
yy3$rnk21 <-  rank(yy3$uprnk.20, ties.method ="average")
yy3$rnk22 <-  rank(yy3$uprnk.21, ties.method ="average")
yy3$rnk23 <-  rank(yy3$uprnk.22, ties.method ="average")
yy3$rnk24 <-  rank(yy3$uprnk.23, ties.method ="average")
yy3$rnk25 <-  rank(yy3$uprnk.24, ties.method ="average")
yy3$rnk26 <-  rank(yy3$uprnk.25, ties.method ="average")
yy3$rnk27 <-  rank(yy3$uprnk.26, ties.method ="average")
yy3$rnk28 <-  rank(yy3$uprnk.27, ties.method ="average")
yy3$rnk29 <-  rank(yy3$uprnk.28, ties.method ="average")

bansh1 <-  function (x) {sum ( ifelse (x < 1001, 1, 0)) }
bansh2 <-  function (x) {sum ( ifelse (x < 2001, 1, 0)) }
bansh3 <-  function (x) {sum ( ifelse (x < 3001, 1, 0)) }
bansh4 <-  function (x) {sum ( ifelse (x < 4001, 1, 0)) }
bansh5 <-  function (x) {sum ( ifelse (x < 5001, 1, 0)) }
bansh6 <-  function (x) {sum ( ifelse (x < 6001, 1, 0)) }
bansh7 <-  function (x) {sum ( ifelse (x < 7001, 1, 0)) }
bansh8 <-  function (x) {sum ( ifelse (x < 8001, 1, 0)) }

yy3$upN1 <- apply(yy3[ , n1:n2], 1, bansh1)
yy3$upN2 <- apply(yy3[ , n1:n2], 1, bansh2)
yy3$upN3 <- apply(yy3[ , n1:n2], 1, bansh3)
yy3$upN4 <- apply(yy3[ , n1:n2], 1, bansh4)
yy3$upN5 <- apply(yy3[ , n1:n2], 1, bansh5)
yy3$upN6 <- apply(yy3[ , n1:n2], 1, bansh6)
yy3$upN7 <- apply(yy3[ , n1:n2], 1, bansh7)
yy3$upN8 <- apply(yy3[ , n1:n2], 1, bansh8)


F1 <- yy3[ , c("Gene.symbol", "upN1", "upN2", "upN3", "upN4", "upN5", "upN6",  "upN7",  "upN8")]

yy3$rnk1  <-  rank(-yy3$uprnk, ties.method ="average")
yy3$rnk2 <-  rank(-yy3$uprnk.1, ties.method ="average")
yy3$rnk3 <-  rank(-yy3$uprnk.2, ties.method ="average")
yy3$rnk4 <-  rank(-yy3$uprnk.3, ties.method ="average")
yy3$rnk5 <-  rank(-yy3$uprnk.4, ties.method ="average")
yy3$rnk6 <-  rank(-yy3$uprnk.5, ties.method ="average")
yy3$rnk7 <-  rank(-yy3$uprnk.6, ties.method ="average")
yy3$rnk8 <-  rank(-yy3$uprnk.7, ties.method ="average")
yy3$rnk9 <-  rank(-yy3$uprnk.8, ties.method ="average")
yy3$rnk10 <-  rank(-yy3$uprnk.9, ties.method ="average")
yy3$rnk11 <-  rank(-yy3$uprnk.10, ties.method ="average")
yy3$rnk12 <-  rank(-yy3$uprnk.11, ties.method ="average")
yy3$rnk13 <-  rank(-yy3$uprnk.12, ties.method ="average")
yy3$rnk14 <-  rank(-yy3$uprnk.13, ties.method ="average")
yy3$rnk15 <-  rank(-yy3$uprnk.14, ties.method ="average")
yy3$rnk16 <-  rank(-yy3$uprnk.15, ties.method ="average")
yy3$rnk17 <-  rank(-yy3$uprnk.16, ties.method ="average")
yy3$rnk18 <-  rank(-yy3$uprnk.17, ties.method ="average")
yy3$rnk19 <-  rank(-yy3$uprnk.18, ties.method ="average")
yy3$rnk20 <-  rank(-yy3$uprnk.19, ties.method ="average")
yy3$rnk21 <-  rank(-yy3$uprnk.20, ties.method ="average")
yy3$rnk22 <-  rank(-yy3$uprnk.21, ties.method ="average")
yy3$rnk23 <-  rank(-yy3$uprnk.22, ties.method ="average")
yy3$rnk24 <-  rank(-yy3$uprnk.23, ties.method ="average")
```

```
yy3$rnk25 <-  rank(-yy3$uprnk.24, ties.method ="average")
yy3$rnk26 <-  rank(-yy3$uprnk.25, ties.method ="average")
yy3$rnk27 <-  rank(-yy3$uprnk.26, ties.method ="average")
yy3$rnk28 <-  rank(-yy3$uprnk.27, ties.method ="average")
yy3$rnk29 <-  rank(-yy3$uprnk.28, ties.method ="average")


bansh1 <-  function (x) {sum ( ifelse (x < 1001, 1, 0)) }
bansh2 <-  function (x) {sum ( ifelse (x < 2001, 1, 0)) }
bansh3 <-  function (x) {sum ( ifelse (x < 3001, 1, 0)) }
bansh4 <-  function (x) {sum ( ifelse (x < 4001, 1, 0)) }
bansh5 <-  function (x) {sum ( ifelse (x < 5001, 1, 0)) }
bansh6 <-  function (x) {sum ( ifelse (x < 6001, 1, 0)) }
bansh7 <-  function (x) {sum ( ifelse (x < 7001, 1, 0)) }
bansh8 <-  function (x) {sum ( ifelse (x < 8001, 1, 0)) }

yy3$dwnN1 <- apply(yy3[ , n1:n2], 1, bansh1)
yy3$dwnN2 <- apply(yy3[ , n1:n2], 1, bansh2)
yy3$dwnN3 <- apply(yy3[ , n1:n2], 1, bansh3)
yy3$dwnN4 <- apply(yy3[ , n1:n2], 1, bansh4)
yy3$dwnN5 <- apply(yy3[ , n1:n2], 1, bansh5)
yy3$dwnN6 <- apply(yy3[ , n1:n2], 1, bansh6)
yy3$dwnN7 <- apply(yy3[ , n1:n2], 1, bansh7)
yy3$dwnN8 <- apply(yy3[ , n1:n2], 1, bansh8)


F2 <- yy3[ , c("Gene.symbol",  "dwnN1", "dwnN2", "dwnN3", "dwnN4", "dwnN5", "dwnN6", "dwnN7", 
"dwnN8")]

zclose <- join(F1, F2, by="Gene.symbol")

zclose$k1 <- zclose$upN1 - zclose$dwnN1
zclose$k2 <- (zclose$upN2 - zclose$dwnN2) * .1
zclose$k3 <- (zclose$upN3 - zclose$dwnN3) * .01
zclose$k4 <- (zclose$upN4 - zclose$dwnN4) * .001
zclose$k5 <- (zclose$upN5 - zclose$dwnN5) * .0001
zclose$k6 <- (zclose$upN6 - zclose$dwnN6) * .00001
zclose$k7 <- (zclose$upN7 - zclose$dwnN7) * .000001
zclose$k8 <- (zclose$upN8 - zclose$dwnN8) * .0000001

zclose$sign1 <- zclose$k1 + zclose$k2 + zclose$k3 + zclose$k4 + zclose$k5 + 
zclose$k6 + zclose$k7 + zclose$k8

zclose$abs <- (abs(zclose$sign1))
zclose2 <- zclose[order (-zclose$abs), ]

write.table(zclose2, file="signinfo.txt", sep="\t",quote=F, col.names=TRUE, row.names=FALSE)

zclose3 <- zclose2[ , c("Gene.symbol", "sign1")]
write.table(zclose3, file="FINAL.txt", sep="\t",quote=F, col.names=TRUE, row.names=FALSE)
```

This text will create a male depression portrait (z2). The output "FINAL.txt" is the same as z2, but has a different name so results can be compared with z2.

Required packages: plyr and dplyr

```
h1 <- read.table ("y1.txt", header = TRUE)
h2 <- read.table ("y2.txt", header = TRUE)
h3 <- read.table ("y5.txt", header = TRUE)
h4 <- read.table ("y6.txt", header = TRUE)
h5 <- read.table ("y7.txt", header = TRUE)
h6 <- read.table ("y8.txt", header = TRUE)
h7 <- read.table ("y9.txt", header = TRUE)
h8 <- read.table ("y11.txt", header = TRUE)
h9 <- read.table ("y12.txt", header = TRUE)
h10 <- read.table ("y13.txt", header = TRUE)
h11 <- read.table ("y14.txt", header = TRUE)
h12 <- read.table ("y15.txt", header = TRUE)
h13 <- read.table ("y16.txt", header = TRUE)
newlist <- list(h1, h2, h3, h4, h5, h6, h7, h8, h9, h10, h11, h12, h13)

hhh <- read.table ("HUGO2.txt", header = TRUE)

ranksup <- function (x) {
x$uprnk <-  rank(-x$sign1, ties.method ="average")
rr1 <- x[ , c (1,3)]
zzz6 <- join(hhh, rr1, by="Gene.symbol")}

gdone <- lapply(newlist, ranksup)

tt <- as.data.frame (gdone)
names (tt) [names (tt) == 'Gene.symbol'] <- 'temp1'
tt3 <- select(tt,-starts_with("Gene"))
names (tt3) [names (tt3) == 'temp1'] <- 'Gene.symbol'

tt3$nacount <- apply(is.na(tt3), 1, sum)

colinfo <- (ncol (tt3)) -2
colinfo1 <- ceiling (colinfo * (1/3))
n1 <- colinfo +3
n2 <- n1 + colinfo -1

yy3 <- tt3 [which (tt3$nacount <= colinfo1) , ]
yy3[is.na(yy3)] <- nrow(yy3)/2

yy3$rnk1  <-  rank(yy3$uprnk, ties.method ="average")
yy3$rnk2 <-  rank(yy3$uprnk.1, ties.method ="average")
yy3$rnk3 <-  rank(yy3$uprnk.2, ties.method ="average")
yy3$rnk4 <-  rank(yy3$uprnk.3, ties.method ="average")
yy3$rnk5 <-  rank(yy3$uprnk.4, ties.method ="average")
yy3$rnk6 <-  rank(yy3$uprnk.5, ties.method ="average")
yy3$rnk7 <-  rank(yy3$uprnk.6, ties.method ="average")
yy3$rnk8 <-  rank(yy3$uprnk.7, ties.method ="average")
yy3$rnk9 <-  rank(yy3$uprnk.8, ties.method ="average")
yy3$rnk10 <-  rank(yy3$uprnk.9, ties.method ="average")
yy3$rnk11 <-  rank(yy3$uprnk.10, ties.method ="average")
yy3$rnk12 <-  rank(yy3$uprnk.11, ties.method ="average")
yy3$rnk13 <-  rank(yy3$uprnk.12, ties.method ="average")

bansh1 <-  function (x) {sum ( ifelse (x < 1001, 1, 0)) }
```

```
bansh2 <-  function (x) {sum ( ifelse (x < 2001, 1, 0)) }
bansh3 <-  function (x) {sum ( ifelse (x < 3001, 1, 0)) }
bansh4 <-  function (x) {sum ( ifelse (x < 4001, 1, 0)) }
bansh5 <-  function (x) {sum ( ifelse (x < 5001, 1, 0)) }
bansh6 <-  function (x) {sum ( ifelse (x < 6001, 1, 0)) }
bansh7 <-  function (x) {sum ( ifelse (x < 7001, 1, 0)) }
bansh8 <-  function (x) {sum ( ifelse (x < 8001, 1, 0)) }

yy3$upN1 <- apply(yy3[ , n1:n2], 1, bansh1)
yy3$upN2 <- apply(yy3[ , n1:n2], 1, bansh2)
yy3$upN3 <- apply(yy3[ , n1:n2], 1, bansh3)
yy3$upN4 <- apply(yy3[ , n1:n2], 1, bansh4)
yy3$upN5 <- apply(yy3[ , n1:n2], 1, bansh5)
yy3$upN6 <- apply(yy3[ , n1:n2], 1, bansh6)
yy3$upN7 <- apply(yy3[ , n1:n2], 1, bansh7)
yy3$upN8 <- apply(yy3[ , n1:n2], 1, bansh8)


F1 <- yy3[ , c("Gene.symbol", "upN1", "upN2", "upN3", "upN4", "upN5", "upN6",  "upN7",  "upN8")]

yy3$rnk1  <-  rank(-yy3$uprnk, ties.method ="average")
yy3$rnk2 <-  rank(-yy3$uprnk.1, ties.method ="average")
yy3$rnk3 <-  rank(-yy3$uprnk.2, ties.method ="average")
yy3$rnk4 <-  rank(-yy3$uprnk.3, ties.method ="average")
yy3$rnk5 <-  rank(-yy3$uprnk.4, ties.method ="average")
yy3$rnk6 <-  rank(-yy3$uprnk.5, ties.method ="average")
yy3$rnk7 <-  rank(-yy3$uprnk.6, ties.method ="average")
yy3$rnk8 <-  rank(-yy3$uprnk.7, ties.method ="average")
yy3$rnk9 <-  rank(-yy3$uprnk.8, ties.method ="average")
yy3$rnk10 <-  rank(-yy3$uprnk.9, ties.method ="average")
yy3$rnk11 <-  rank(-yy3$uprnk.10, ties.method ="average")
yy3$rnk12 <-  rank(-yy3$uprnk.11, ties.method ="average")
yy3$rnk13 <-  rank(-yy3$uprnk.12, ties.method ="average")


bansh1 <-  function (x) {sum ( ifelse (x < 1001, 1, 0)) }
bansh2 <-  function (x) {sum ( ifelse (x < 2001, 1, 0)) }
bansh3 <-  function (x) {sum ( ifelse (x < 3001, 1, 0)) }
bansh4 <-  function (x) {sum ( ifelse (x < 4001, 1, 0)) }
bansh5 <-  function (x) {sum ( ifelse (x < 5001, 1, 0)) }
bansh6 <-  function (x) {sum ( ifelse (x < 6001, 1, 0)) }
bansh7 <-  function (x) {sum ( ifelse (x < 7001, 1, 0)) }
bansh8 <-  function (x) {sum ( ifelse (x < 8001, 1, 0)) }

yy3$dwnN1 <- apply(yy3[ , n1:n2], 1, bansh1)
yy3$dwnN2 <- apply(yy3[ , n1:n2], 1, bansh2)
yy3$dwnN3 <- apply(yy3[ , n1:n2], 1, bansh3)
yy3$dwnN4 <- apply(yy3[ , n1:n2], 1, bansh4)
yy3$dwnN5 <- apply(yy3[ , n1:n2], 1, bansh5)
yy3$dwnN6 <- apply(yy3[ , n1:n2], 1, bansh6)
yy3$dwnN7 <- apply(yy3[ , n1:n2], 1, bansh7)
yy3$dwnN8 <- apply(yy3[ , n1:n2], 1, bansh8)


F2 <- yy3[ , c("Gene.symbol",  "dwnN1", "dwnN2", "dwnN3", "dwnN4", "dwnN5", "dwnN6", "dwnN7", 
"dwnN8")]

zclose <- join(F1, F2, by="Gene.symbol")

zclose$k1 <- zclose$upN1 - zclose$dwnN1
zclose$k2 <- (zclose$upN2 - zclose$dwnN2) * .1
zclose$k3 <- (zclose$upN3 - zclose$dwnN3) * .01
zclose$k4 <- (zclose$upN4 - zclose$dwnN4) * .001
```

```
zclose$k5 <- (zclose$upN5 - zclose$dwnN5) * .0001
zclose$k6 <- (zclose$upN6 - zclose$dwnN6) * .00001
zclose$k7 <- (zclose$upN7 - zclose$dwnN7) * .000001
zclose$k8 <- (zclose$upN8 - zclose$dwnN8) * .0000001

zclose$sign1 <- zclose$k1 + zclose$k2 + zclose$k3 + zclose$k4 + zclose$k5 +
zclose$k6 + zclose$k7 + zclose$k8


zclose$abs <- (abs(zclose$sign1))
zclose2 <- zclose[order (-zclose$abs), ]

write.table(zclose2, file="signinfo.txt", sep="\t",quote=F, col.names=TRUE, row.names=FALSE)

zclose3 <- zclose2[ , c("Gene.symbol", "sign1")]
write.table(zclose3, file="FINAL.txt", sep="\t",quote=F, col.names=TRUE, row.names=FALSE)
```

This text will create a female depression portrait (z3). The output "FINAL.txt" is the same as z3, but has a different name so results can be compared with z3.

Required packages: plyr and dplyr

```
h1 <- read.table ("x1.txt", header = TRUE)
h2 <- read.table ("x2.txt", header = TRUE)
h3 <- read.table ("x5.txt", header = TRUE)
h4 <- read.table ("x6.txt", header = TRUE)
h5 <- read.table ("x7.txt", header = TRUE)
h6 <- read.table ("x8.txt", header = TRUE)
h7 <- read.table ("x9.txt", header = TRUE)
h8 <- read.table ("x10.txt", header = TRUE)
h9 <- read.table ("x11.txt", header = TRUE)
h10 <- read.table ("x12.txt", header = TRUE)
h11 <- read.table ("x13.txt", header = TRUE)
h12 <- read.table ("x14.txt", header = TRUE)
h13 <- read.table ("x15.txt", header = TRUE)


newlist <- list(h1, h2, h3, h4, h5, h6, h7, h8, h9, h10, h11, h12, h13)

hhh <- read.table ("HUGO2.txt", header = TRUE)

ranksup <- function (x) {
x$uprnk <-  rank(-x$sign1, ties.method ="average")
rr1 <- x[ , c (1,3)]
zzz6 <- join(hhh, rr1, by="Gene.symbol")}

gdone <- lapply(newlist, ranksup)

tt <- as.data.frame (gdone)
names (tt) [names (tt) == 'Gene.symbol'] <- 'temp1'
tt3 <- select(tt,-starts_with("Gene"))
names (tt3) [names (tt3) == 'temp1'] <- 'Gene.symbol'

tt3$nacount <- apply(is.na(tt3), 1, sum)

colinfo <- (ncol (tt3)) -2
colinfo1 <- ceiling (colinfo * (1/3))
n1 <- colinfo +3
n2 <- n1 + colinfo -1

yy3 <- tt3 [which (tt3$nacount <= colinfo1) , ]
yy3[is.na(yy3)] <- nrow(yy3)/2


yy3$rnk1  <-  rank(yy3$uprnk, ties.method ="average")
yy3$rnk2 <-  rank(yy3$uprnk.1, ties.method ="average")
yy3$rnk3 <-  rank(yy3$uprnk.2, ties.method ="average")
yy3$rnk4 <-  rank(yy3$uprnk.3, ties.method ="average")
yy3$rnk5 <-  rank(yy3$uprnk.4, ties.method ="average")
yy3$rnk6 <-  rank(yy3$uprnk.5, ties.method ="average")
yy3$rnk7 <-  rank(yy3$uprnk.6, ties.method ="average")
yy3$rnk8 <-  rank(yy3$uprnk.7, ties.method ="average")
yy3$rnk9 <-  rank(yy3$uprnk.8, ties.method ="average")
yy3$rnk10 <-  rank(yy3$uprnk.9, ties.method ="average")
yy3$rnk11 <-  rank(yy3$uprnk.10, ties.method ="average")
yy3$rnk12 <-  rank(yy3$uprnk.11, ties.method ="average")
yy3$rnk13 <-  rank(yy3$uprnk.12, ties.method ="average")

bansh1 <-  function (x) {sum ( ifelse (x < 1001, 1, 0)) }
bansh2 <-  function (x) {sum ( ifelse (x < 2001, 1, 0)) }
bansh3 <-  function (x) {sum ( ifelse (x < 3001, 1, 0)) }
```

```
bansh4 <-  function (x) {sum ( ifelse (x < 4001, 1, 0)) }
bansh5 <-  function (x) {sum ( ifelse (x < 5001, 1, 0)) }
bansh6 <-  function (x) {sum ( ifelse (x < 6001, 1, 0)) }
bansh7 <-  function (x) {sum ( ifelse (x < 7001, 1, 0)) }
bansh8 <-  function (x) {sum ( ifelse (x < 8001, 1, 0)) }

yy3$upN1 <- apply(yy3[ , n1:n2], 1, bansh1)
yy3$upN2 <- apply(yy3[ , n1:n2], 1, bansh2)
yy3$upN3 <- apply(yy3[ , n1:n2], 1, bansh3)
yy3$upN4 <- apply(yy3[ , n1:n2], 1, bansh4)
yy3$upN5 <- apply(yy3[ , n1:n2], 1, bansh5)
yy3$upN6 <- apply(yy3[ , n1:n2], 1, bansh6)
yy3$upN7 <- apply(yy3[ , n1:n2], 1, bansh7)
yy3$upN8 <- apply(yy3[ , n1:n2], 1, bansh8)


F1 <- yy3[ , c("Gene.symbol", "upN1", "upN2", "upN3", "upN4", "upN5", "upN6",  "upN7",  "upN8")]

yy3$rnk1  <-  rank(-yy3$uprnk, ties.method ="average")
yy3$rnk2 <-  rank(-yy3$uprnk.1, ties.method ="average")
yy3$rnk3 <-  rank(-yy3$uprnk.2, ties.method ="average")
yy3$rnk4 <-  rank(-yy3$uprnk.3, ties.method ="average")
yy3$rnk5 <-  rank(-yy3$uprnk.4, ties.method ="average")
yy3$rnk6 <-  rank(-yy3$uprnk.5, ties.method ="average")
yy3$rnk7 <-  rank(-yy3$uprnk.6, ties.method ="average")
yy3$rnk8 <-  rank(-yy3$uprnk.7, ties.method ="average")
yy3$rnk9 <-  rank(-yy3$uprnk.8, ties.method ="average")
yy3$rnk10 <-  rank(-yy3$uprnk.9, ties.method ="average")
yy3$rnk11 <-  rank(-yy3$uprnk.10, ties.method ="average")
yy3$rnk12 <-  rank(-yy3$uprnk.11, ties.method ="average")
yy3$rnk13 <-  rank(-yy3$uprnk.12, ties.method ="average")


bansh1 <-  function (x) {sum ( ifelse (x < 1001, 1, 0)) }
bansh2 <-  function (x) {sum ( ifelse (x < 2001, 1, 0)) }
bansh3 <-  function (x) {sum ( ifelse (x < 3001, 1, 0)) }
bansh4 <-  function (x) {sum ( ifelse (x < 4001, 1, 0)) }
bansh5 <-  function (x) {sum ( ifelse (x < 5001, 1, 0)) }
bansh6 <-  function (x) {sum ( ifelse (x < 6001, 1, 0)) }
bansh7 <-  function (x) {sum ( ifelse (x < 7001, 1, 0)) }
bansh8 <-  function (x) {sum ( ifelse (x < 8001, 1, 0)) }

yy3$dwnN1 <- apply(yy3[ , n1:n2], 1, bansh1)
yy3$dwnN2 <- apply(yy3[ , n1:n2], 1, bansh2)
yy3$dwnN3 <- apply(yy3[ , n1:n2], 1, bansh3)
yy3$dwnN4 <- apply(yy3[ , n1:n2], 1, bansh4)
yy3$dwnN5 <- apply(yy3[ , n1:n2], 1, bansh5)
yy3$dwnN6 <- apply(yy3[ , n1:n2], 1, bansh6)
yy3$dwnN7 <- apply(yy3[ , n1:n2], 1, bansh7)
yy3$dwnN8 <- apply(yy3[ , n1:n2], 1, bansh8)


F2 <- yy3[ , c("Gene.symbol",  "dwnN1", "dwnN2", "dwnN3", "dwnN4", "dwnN5", "dwnN6", "dwnN7",
"dwnN8")]

zclose <- join(F1, F2, by="Gene.symbol")

zclose$k1 <- zclose$upN1 - zclose$dwnN1
zclose$k2 <- (zclose$upN2 - zclose$dwnN2) * .1
zclose$k3 <- (zclose$upN3 - zclose$dwnN3) * .01
zclose$k4 <- (zclose$upN4 - zclose$dwnN4) * .001
zclose$k5 <- (zclose$upN5 - zclose$dwnN5) * .0001
```

```
zclose$k6 <- (zclose$upN6 - zclose$dwnN6) * .00001
zclose$k7 <- (zclose$upN7 - zclose$dwnN7) * .000001
zclose$k8 <- (zclose$upN8 - zclose$dwnN8) * .0000001

zclose$sign1 <- zclose$k1 + zclose$k2 + zclose$k3 + zclose$k4 + zclose$k5 +
zclose$k6 + zclose$k7 + zclose$k8


zclose$abs <- (abs(zclose$sign1))
zclose2 <- zclose[order (-zclose$abs), ]

write.table(zclose2, file="signinfo.txt", sep="\t",quote=F, col.names=TRUE, row.names=FALSE)

zclose3 <- zclose2[ , c("Gene.symbol", "sign1")]
write.table(zclose3, file="FINAL.txt", sep="\t",quote=F, col.names=TRUE, row.names=FALSE)
```

This script allows one to compare any individual dataset (e.g., “z1”, the combined portrait) against all others. The outputs include pvalues and -log10 of pvalue for each comparison: genes moving in the same direction (A=up/up; D=down/down) and in opposite directions (B=up treatment/down portrait; C= down treatment/up portrait). The final score can be calculated in Excel by adding the scores of A and D (same direction) and subtracting the scores of B and C (opposite direction) for each comparison.

```
gg <- read.table ("z1.txt", header = TRUE)

h1 <- read.table ("x1.txt", header = TRUE)
h2 <- read.table ("x2.txt", header = TRUE)
h3 <- read.table ("x3.txt", header = TRUE)
h4 <- read.table ("x4.txt", header = TRUE)
h5 <- read.table ("x5.txt", header = TRUE)
h6 <- read.table ("x6.txt", header = TRUE)
h7 <- read.table ("x7.txt", header = TRUE)
h8 <- read.table ("x8.txt", header = TRUE)
h9 <- read.table ("x9.txt", header = TRUE)
h10 <- read.table ("x10.txt", header = TRUE)
h11 <- read.table ("x11.txt", header = TRUE)
h12 <- read.table ("x12.txt", header = TRUE)
h13 <- read.table ("x13.txt", header = TRUE)
h14 <- read.table ("x14.txt", header = TRUE)
h15 <- read.table ("x15.txt", header = TRUE)
h16 <- read.table ("y1.txt", header = TRUE)
h17 <- read.table ("y2.txt", header = TRUE)
h18 <- read.table ("y3.txt", header = TRUE)
h19 <- read.table ("y4.txt", header = TRUE)
h20 <- read.table ("y5.txt", header = TRUE)
h21 <- read.table ("y6.txt", header = TRUE)
h22 <- read.table ("y7.txt", header = TRUE)
h23 <- read.table ("y8.txt", header = TRUE)
h24 <- read.table ("y9.txt", header = TRUE)
h25 <- read.table ("y10.txt", header = TRUE)
h26 <- read.table ("y11.txt", header = TRUE)
h27 <- read.table ("y12.txt", header = TRUE)
h28 <- read.table ("y13.txt", header = TRUE)
h29 <- read.table ("y14.txt", header = TRUE)
h30 <- read.table ("y15.txt", header = TRUE)
h31 <- read.table ("y16.txt", header = TRUE)
h32 <- read.table ("xy1.txt", header = TRUE)
h33 <- read.table ("xy2.txt", header = TRUE)
h34 <- read.table ("xy3.txt", header = TRUE)
h35 <- read.table ("xy4.txt", header = TRUE)
h36 <- read.table ("z1.txt", header = TRUE)
h37 <- read.table ("z2.txt", header = TRUE)
h38 <- read.table ("z3.txt", header = TRUE)

newlist <- list(h1, h2, h3, h4, h5, h6, h7, h8, h9, h10, h11, h12, h13, h14, h15, h16, h17, h18, h19, h20, h21, h22, h23, h24, h25, h26, h27, h28, h29, h30,
h31, h32, h33, h34, h35, h36, h37, h38)


matches1 <- function (x) {
zzz <- merge(gg,x,by="Gene.symbol")
order.scores <- order(zzz$sign1.x)
dat1 <- zzz[order.scores,]
dat1$rank <- rank(-dat1$sign1.x, ties.method= "average")
order.scores1 <- order(dat1$sign1.y)
dat2 <- dat1[order.scores1,]
dat2$rank2 <- rank(-dat2$sign1.y, ties.method= "average")
mmm<- dat2[ ,c(1,4,5)]
colnames(mmm) <- c("Gene.symbol", "r1","r2")
mmmA <- mmm[order (mmm$r1),]
zz <- nrow (mmmA)
q <- mmmA[1:1000, 3]
qA <- sum(ifelse(q <1001, 1, 0))
```

```
qC <- sum(ifelse (q > zz-1001, 1, 0))
mmmB <- mmm[order (-mmm$r1),]
q1 <- mmmB[1:1000, 3]
qB <- sum(ifelse(q1 <1001, 1, 0))
qD <- sum(ifelse (q1 > zz-1001, 1, 0))
hA <- phyper(qA,1000,zz-1000,1000, lower.tail = F)
hB <- phyper(qB,1000,zz-1000,1000, lower.tail = F)
hC <- phyper(qC,1000, zz-1000,1000, lower.tail = F)
hD <- phyper(qD,1000, zz-1000,1000, lower.tail = F)
sst1 <- data.frame (GroupA = hA)
sst2 <- data.frame (GroupB = hB)
sst3 <- data.frame (GroupC = hC)
sst4 <- data.frame (GroupD = hD)
write.table (sst1, file="pvalueA.txt", quote=F, sep="\t", col.names=FALSE, row.names=FALSE, append = TRUE)
write.table (sst2, file="pvalueB.txt", quote=F, sep="\t", col.names=FALSE, row.names=FALSE, append = TRUE)
write.table (sst3, file="pvalueC.txt", quote=F, sep="\t", col.names=FALSE, row.names=FALSE, append = TRUE)
write.table (sst4, file="pvalueD.txt", quote=F, sep="\t", col.names=FALSE, row.names=FALSE, append = TRUE)
sA <- log10(hA)*-1
sB <- log10(hB)*-1
sC <- log10(hC)*-1
sD <- log10(hD)*-1
s1st1 <- data.frame (GroupA = sA)
s1st2 <- data.frame (GroupB = sB)
s1st3 <- data.frame (GroupC = sC)
s1st4 <- data.frame (GroupD = sD)
write.table (s1st1, file="log10A.txt", quote=F, sep="\t", col.names=FALSE, row.names=FALSE, append = TRUE)
write.table (s1st2, file="log10B.txt", quote=F, sep="\t", col.names=FALSE, row.names=FALSE, append = TRUE)
write.table (s1st3, file="log10C.txt", quote=F, sep="\t", col.names=FALSE, row.names=FALSE, append = TRUE)
write.table (s1st4, file="log10D.txt", quote=F, sep="\t", col.names=FALSE, row.names=FALSE, append = TRUE)
}

gdone <- lapply(newlist, matches1)
```

This text compares all 200+ treatments against a given portrait (e.g., z1). The outputs include pvalues and -log10 of pvalue for each comparison: genes moving in the same direction (A=up/up; D=down/down) and in opposite directions (B=up treatment/down portrait; C= down treatment/up portrait). The outputs also include counts of genes for A, B, C, and D and the size of the 'universe' (total number of common genes between two lists that is used for the hypergeometric tests). The final score can be calculated in Excel by adding the scores of B and C and subtracting the scores of A and D for each comparison. All files are from publicly available datasets and the files referred to here are available upon request.

```
ggg <- read.table ("z1.txt", header = TRUE)
h1 <- read.table ("t1.txt", header = TRUE)
h2 <- read.table ("t2.txt", header = TRUE)
h3 <- read.table ("t3.txt", header = TRUE)
h4 <- read.table ("t4.txt", header = TRUE)
h5 <- read.table ("t5.txt", header = TRUE)
h6 <- read.table ("t6.txt", header = TRUE)
h7 <- read.table ("t7.txt", header = TRUE)
h8 <- read.table ("t8.txt", header = TRUE)
h9 <- read.table ("t9.txt", header = TRUE)
h10 <- read.table ("t10.txt", header = TRUE)
h11 <- read.table ("t11.txt", header = TRUE)
h12 <- read.table ("t12.txt", header = TRUE)
h13 <- read.table ("t13.txt", header = TRUE)
h14 <- read.table ("t14.txt", header = TRUE)
h15 <- read.table ("t15.txt", header = TRUE)
h16 <- read.table ("t16.txt", header = TRUE)
h17 <- read.table ("t17.txt", header = TRUE)
h18 <- read.table ("t18.txt", header = TRUE)
h19 <- read.table ("t19.txt", header = TRUE)
h20 <- read.table ("t20.txt", header = TRUE)
h21 <- read.table ("t21.txt", header = TRUE)
h22 <- read.table ("t22.txt", header = TRUE)
h23 <- read.table ("t23.txt", header = TRUE)
h24 <- read.table ("t24.txt", header = TRUE)
h25 <- read.table ("t25.txt", header = TRUE)
h26 <- read.table ("t26.txt", header = TRUE)
h27 <- read.table ("t27.txt", header = TRUE)
h28 <- read.table ("t28.txt", header = TRUE)
h29 <- read.table ("t29.txt", header = TRUE)
h30 <- read.table ("t30.txt", header = TRUE)
h31 <- read.table ("t31.txt", header = TRUE)
h32 <- read.table ("t32.txt", header = TRUE)
h33 <- read.table ("t33.txt", header = TRUE)
h34 <- read.table ("t34.txt", header = TRUE)
h35 <- read.table ("t35.txt", header = TRUE)
h36 <- read.table ("t36.txt", header = TRUE)
h37 <- read.table ("t37.txt", header = TRUE)
h38 <- read.table ("t38.txt", header = TRUE)
h39 <- read.table ("t39.txt", header = TRUE)
h40 <- read.table ("t40.txt", header = TRUE)
h41 <- read.table ("t41.txt", header = TRUE)
h42 <- read.table ("t42.txt", header = TRUE)
h43 <- read.table ("t43.txt", header = TRUE)
h44 <- read.table ("t44.txt", header = TRUE)
h45 <- read.table ("t45.txt", header = TRUE)
h46 <- read.table ("t46.txt", header = TRUE)
h47 <- read.table ("t47.txt", header = TRUE)
h48 <- read.table ("t48.txt", header = TRUE)
h49 <- read.table ("t49.txt", header = TRUE)
h50 <- read.table ("t50.txt", header = TRUE)
h51 <- read.table ("t51.txt", header = TRUE)
h52 <- read.table ("t52.txt", header = TRUE)
h53 <- read.table ("t53.txt", header = TRUE)
h54 <- read.table ("t54.txt", header = TRUE)
h55 <- read.table ("t55.txt", header = TRUE)
h56 <- read.table ("t56.txt", header = TRUE)
h57 <- read.table ("t57.txt", header = TRUE)
h58 <- read.table ("t58.txt", header = TRUE)
h59 <- read.table ("t59.txt", header = TRUE)
h60 <- read.table ("t60.txt", header = TRUE)
h61 <- read.table ("t61.txt", header = TRUE)
h62 <- read.table ("t62.txt", header = TRUE)
h63 <- read.table ("t63.txt", header = TRUE)
h64 <- read.table ("t64.txt", header = TRUE)
h65 <- read.table ("t65.txt", header = TRUE)
h66 <- read.table ("t66.txt", header = TRUE)
h67 <- read.table ("t67.txt", header = TRUE)
h68 <- read.table ("t68.txt", header = TRUE)
h69 <- read.table ("t69.txt", header = TRUE)
h70 <- read.table ("t70.txt", header = TRUE)
h71 <- read.table ("t71.txt", header = TRUE)
h72 <- read.table ("t72.txt", header = TRUE)
h73 <- read.table ("t73.txt", header = TRUE)
h74 <- read.table ("t74.txt", header = TRUE)
h75 <- read.table ("t75.txt", header = TRUE)
h76 <- read.table ("t76.txt", header = TRUE)
h77 <- read.table ("t77.txt", header = TRUE)
h78 <- read.table ("t78.txt", header = TRUE)
h79 <- read.table ("t79.txt", header = TRUE)
h80 <- read.table ("t80.txt", header = TRUE)
h81 <- read.table ("t81.txt", header = TRUE)
h82 <- read.table ("t82.txt", header = TRUE)
h83 <- read.table ("t83.txt", header = TRUE)
h84 <- read.table ("t84.txt", header = TRUE)
h85 <- read.table ("t85.txt", header = TRUE)
h86 <- read.table ("t86.txt", header = TRUE)
h87 <- read.table ("t87.txt", header = TRUE)
h88 <- read.table ("t88.txt", header = TRUE)
h89 <- read.table ("t89.txt", header = TRUE)
```

```
h90 <- read.table ("t90.txt", header = TRUE)
h91 <- read.table ("t91.txt", header = TRUE)
h92 <- read.table ("t92.txt", header = TRUE)
h93 <- read.table ("t93.txt", header = TRUE)
h94 <- read.table ("t94.txt", header = TRUE)
h95 <- read.table ("t95.txt", header = TRUE)
h96 <- read.table ("t96.txt", header = TRUE)
h97 <- read.table ("t97.txt", header = TRUE)
h98 <- read.table ("t98.txt", header = TRUE)
h99 <- read.table ("t99.txt", header = TRUE)
h100 <- read.table ("t100.txt", header = TRUE)
h101 <- read.table ("t101.txt", header = TRUE)
h102 <- read.table ("t102.txt", header = TRUE)
h103 <- read.table ("t103.txt", header = TRUE)
h104 <- read.table ("t104.txt", header = TRUE)
h105 <- read.table ("t105.txt", header = TRUE)
h106 <- read.table ("t106.txt", header = TRUE)
h107 <- read.table ("t107.txt", header = TRUE)
h108 <- read.table ("t108.txt", header = TRUE)
h109 <- read.table ("t109.txt", header = TRUE)
h110 <- read.table ("t110.txt", header = TRUE)
h111 <- read.table ("t111.txt", header = TRUE)
h112 <- read.table ("t112.txt", header = TRUE)
h113 <- read.table ("t113.txt", header = TRUE)
h114 <- read.table ("t114.txt", header = TRUE)
h115 <- read.table ("t115.txt", header = TRUE)
h116 <- read.table ("t116.txt", header = TRUE)
h117 <- read.table ("t117.txt", header = TRUE)
h118 <- read.table ("t118.txt", header = TRUE)
h119 <- read.table ("t119.txt", header = TRUE)
h120 <- read.table ("t120.txt", header = TRUE)
h121 <- read.table ("t121.txt", header = TRUE)
h122 <- read.table ("t122.txt", header = TRUE)
h123 <- read.table ("t123.txt", header = TRUE)
h124 <- read.table ("t124.txt", header = TRUE)
h125 <- read.table ("t125.txt", header = TRUE)
h126 <- read.table ("t126.txt", header = TRUE)
h127 <- read.table ("t127.txt", header = TRUE)
h128 <- read.table ("t128.txt", header = TRUE)
h129 <- read.table ("t129.txt", header = TRUE)
h130 <- read.table ("t130.txt", header = TRUE)
h131 <- read.table ("t131.txt", header = TRUE)
h132 <- read.table ("t132.txt", header = TRUE)
h133 <- read.table ("t133.txt", header = TRUE)
h134 <- read.table ("t134.txt", header = TRUE)
h135 <- read.table ("t135.txt", header = TRUE)
h136 <- read.table ("t136.txt", header = TRUE)
h137 <- read.table ("t137.txt", header = TRUE)
h138 <- read.table ("t138.txt", header = TRUE)
h139 <- read.table ("t139.txt", header = TRUE)
h140 <- read.table ("t140.txt", header = TRUE)
h141 <- read.table ("t141.txt", header = TRUE)
h142 <- read.table ("t142.txt", header = TRUE)
h143 <- read.table ("t143.txt", header = TRUE)
h144 <- read.table ("t144.txt", header = TRUE)
h145 <- read.table ("t145.txt", header = TRUE)
h146 <- read.table ("t146.txt", header = TRUE)
h147 <- read.table ("t147.txt", header = TRUE)
h148 <- read.table ("t148.txt", header = TRUE)
h149 <- read.table ("t149.txt", header = TRUE)
h150 <- read.table ("t150.txt", header = TRUE)
h151 <- read.table ("t151.txt", header = TRUE)
h152 <- read.table ("t152.txt", header = TRUE)
h153 <- read.table ("t153.txt", header = TRUE)
h154 <- read.table ("t154.txt", header = TRUE)
h155 <- read.table ("t155.txt", header = TRUE)
h156 <- read.table ("t156.txt", header = TRUE)
h157 <- read.table ("t157.txt", header = TRUE)
h158 <- read.table ("t158.txt", header = TRUE)
h159 <- read.table ("t159.txt", header = TRUE)
h160 <- read.table ("t160.txt", header = TRUE)
h161 <- read.table ("t161.txt", header = TRUE)
h162 <- read.table ("t162.txt", header = TRUE)
h163 <- read.table ("t163.txt", header = TRUE)
h164 <- read.table ("t164.txt", header = TRUE)
h165 <- read.table ("t165.txt", header = TRUE)
h166 <- read.table ("t166.txt", header = TRUE)
h167 <- read.table ("t167.txt", header = TRUE)
h168 <- read.table ("t168.txt", header = TRUE)
h169 <- read.table ("t169.txt", header = TRUE)
h170 <- read.table ("t170.txt", header = TRUE)
h171 <- read.table ("t171.txt", header = TRUE)
h172 <- read.table ("t172.txt", header = TRUE)
h173 <- read.table ("t173.txt", header = TRUE)
h174 <- read.table ("t174.txt", header = TRUE)
h175 <- read.table ("t175.txt", header = TRUE)
h176 <- read.table ("t176.txt", header = TRUE)
h177 <- read.table ("t177.txt", header = TRUE)
h178 <- read.table ("t178.txt", header = TRUE)
h179 <- read.table ("t179.txt", header = TRUE)
h180 <- read.table ("t180.txt", header = TRUE)
h181 <- read.table ("t181.txt", header = TRUE)
h182 <- read.table ("t182.txt", header = TRUE)
h183 <- read.table ("t183.txt", header = TRUE)
h184 <- read.table ("t184.txt", header = TRUE)
h185 <- read.table ("t185.txt", header = TRUE)
h186 <- read.table ("t186.txt", header = TRUE)
h187 <- read.table ("t187.txt", header = TRUE)
h188 <- read.table ("t188.txt", header = TRUE)
h189 <- read.table ("t189.txt", header = TRUE)
h190 <- read.table ("t190.txt", header = TRUE)
h191 <- read.table ("t191.txt", header = TRUE)
h192 <- read.table ("t192.txt", header = TRUE)
h193 <- read.table ("t193.txt", header = TRUE)
h194 <- read.table ("t194.txt", header = TRUE)
h195 <- read.table ("t195.txt", header = TRUE)
h196 <- read.table ("t196.txt", header = TRUE)
h197 <- read.table ("t197.txt", header = TRUE)
h198 <- read.table ("t198.txt", header = TRUE)
h199 <- read.table ("t199.txt", header = TRUE)
```

```
h200 <- read.table ("t200.txt", header = TRUE)
h201 <- read.table ("t201.txt", header = TRUE)
h202 <- read.table ("t202.txt", header = TRUE)
h203 <- read.table ("t203.txt", header = TRUE)
h204 <- read.table ("t204.txt", header = TRUE)
h205 <- read.table ("t205.txt", header = TRUE)

newlist <- list(h1, h2, h3, h4, h5, h6, h7, h8, h9, h10, h11, h12, h13, h14,
    h15, h16, h17, h18, h19, h20, h21, h22, h23, h24, h25, h26, h27,
    h28, h29, h30, h31, h32, h33, h34, h35, h36, h37, h38, h39, h40,
    h41, h42, h43, h44, h45, h46, h47, h48, h49, h50, h51, h52, h53,
    h54, h55, h56, h57, h58, h59, h60, h61, h62, h63, h64, h65, h66,
    h67, h68, h69, h70, h71, h72, h73, h74, h75, h76, h77, h78, h79,
    h80, h81, h82, h83, h84, h85, h86, h87, h88, h89, h90, h91, h92,
    h93, h94, h95, h96, h97, h98, h99, h100, h101, h102, h103, h104, h105,
    h106, h107, h108, h109, h110, h111, h112, h113, h114, h115, h116, h117, h118,
    h119, h120, h121, h122, h123, h124, h125, h126, h127, h128, h129, h130, h131,
    h132, h133, h134, h135, h136, h137, h138, h139, h140, h141, h142, h143, h144,
    h145, h146, h147, h148, h149, h150, h151, h152, h153, h154, h155, h156, h157,
    h158, h159, h160, h161, h162, h163, h164, h165, h166, h167, h168, h169, h170,
    h171, h172, h173, h174, h175, h176, h177, h178, h179, h180, h181, h182, h183,
    h184, h185, h186, h187, h188, h189, h190, h191, h192, h193, h194, h195, h196,
    h197, h198, h199, h200, h201, h202, h203, h204, h205)

matches1 <- function (x) {
zzz <- merge(ggg,x,by="Gene.symbol")
zzz1 <- zzz[complete.cases(zzz[ , 3]),]
order.scores <- order(zzz1$sign1.x)
dat1 <- zzz1[order.scores,]
dat1$rank <- rank(-dat1$sign1.x, ties.method= "average")
order.scores1 <- order(dat1$sign1.y)
dat2 <- dat1[order.scores1,]
dat2$rank2 <- rank(-dat2$sign1.y, ties.method= "average")
mmm<- dat2[ ,c(1,4,5)]
colnames(mmm) <- c("Gene.symbol", "r1","r2")
mmmA <- mmm[order (mmm$r1),]
zz <- nrow (mmmA)
q <- mmmA[1:1000, 3]
qA <- sum(ifelse(q <1001, 1, 0))
qC <- sum(ifelse (q > zz-1001, 1, 0))
mmmB <- mmm[order (-mmm$r1),]
q1 <- mmmB[1:1000, 3]
qB <- sum(ifelse(q1 <1001, 1, 0))
qD <- sum(ifelse (q1 > zz-1001, 1, 0))
hA <- phyper(qA,1000,zz-1000,1000, lower.tail = F)
hB <- phyper(qB,1000,zz-1000,1000, lower.tail = F)
hC <- phyper(qC,1000, zz-1000,1000, lower.tail = F)
hD <- phyper(qD,1000, zz-1000,1000, lower.tail = F)
sst1 <- data.frame (GroupA = hA)
sst2 <- data.frame (GroupB = hB)
sst3 <- data.frame (GroupC = hC)
sst4 <- data.frame (GroupD = hD)
write.table (sst1, file="pvalueA.txt", quote=F, sep="\t", col.names=FALSE, row.names=FALSE, append = TRUE)
write.table (sst2, file="pvalueB.txt", quote=F, sep="\t", col.names=FALSE, row.names=FALSE, append = TRUE)
write.table (sst3, file="pvalueC.txt", quote=F, sep="\t", col.names=FALSE, row.names=FALSE, append = TRUE)
write.table (sst4, file="pvalueD.txt", quote=F, sep="\t", col.names=FALSE, row.names=FALSE, append = TRUE)
sA <- log10(hA)*-1
sB <- log10(hB)*-1
sC <- log10(hC)*-1
sD <- log10(hD)*-1
s1st1 <- data.frame (GroupA = sA)
s1st2 <- data.frame (GroupB = sB)
s1st3 <- data.frame (GroupC = sC)
s1st4 <- data.frame (GroupD = sD)
write.table (s1st1, file="log10A.txt", quote=F, sep="\t", col.names=FALSE, row.names=FALSE, append = TRUE)
write.table (s1st2, file="log10B.txt", quote=F, sep="\t", col.names=FALSE, row.names=FALSE, append = TRUE)
write.table (s1st3, file="log10C.txt", quote=F, sep="\t", col.names=FALSE, row.names=FALSE, append = TRUE)
write.table (s1st4, file="log10D.txt", quote=F, sep="\t", col.names=FALSE, row.names=FALSE, append = TRUE)
write.table (qA, file="countA.txt", quote=F, sep="\t", col.names=FALSE, row.names=FALSE, append = TRUE)
write.table (qB, file="countB.txt", quote=F, sep="\t", col.names=FALSE, row.names=FALSE, append = TRUE)
write.table (qC, file="countC.txt", quote=F, sep="\t", col.names=FALSE, row.names=FALSE, append = TRUE)
write.table (qD, file="countD.txt", quote=F, sep="\t", col.names=FALSE, row.names=FALSE, append = TRUE)
write.table (zz, file="universe.txt", quote=F, sep="\t", col.names=FALSE, row.names=FALSE, append = TRUE)
}

gdone <- lapply(newlist, matches1)
```

Compare any two lists. The text below allows one to identify genes moving in the same direction (A=up/up; D=down/down) and in opposite directions (B=up list1/down list2; C= down list1/up list2). Output “twithz.txt” can be uploaded online into RRHO for analysis.

```
hhh <- read.table ("t34.txt", header = TRUE)
ggg <- read.table ("z1.txt", header = TRUE)

zzz <- merge(ggg,hhh,by="Gene.symbol")
zzz1 <- zzz[complete.cases(zzz[ , 3]),]
order.scores <- order(zzz1$sign1.x)
dat1 <- zzz1[order.scores,]
dat1$rank <- rank(-dat1$sign1.x, ties.method= "random")
order.scores1 <- order(dat1$sign1.y)
dat2 <- dat1[order.scores1,]
dat2$rank2 <- rank(-dat2$sign1.y, ties.method= "random")
mmm<- dat2[ ,c(1,4,5)]
colnames(mmm) <- c("Gene.symbol", "r1","r2")
mmmA <- mmm[order (mmm$r1),]
zz <- nrow (mmmA)
q <- mmmA[1:1000, 3]
qA <- sum(ifelse(q <1001, 1, 0))
qC <- sum(ifelse (q > zz-1001, 1, 0))
mmmB <- mmm[order (-mmm$r1),]
q1 <- mmmB[1:1000, 3]
qB <- sum(ifelse(q1 <1001, 1, 0))
qD <- sum(ifelse (q1 > zz-1001, 1, 0))
hA <- phyper(qA,1000,zz-1000,1000, lower.tail = F)
hB <- phyper(qB,1000,zz-1000,1000, lower.tail = F)
hC <- phyper(qC,1000, zz-1000,1000, lower.tail = F)
hD <- phyper(qD,1000, zz-1000,1000, lower.tail = F)
sA <- ifelse(q <1001, as.character(mmmA$Gene.symbol), NA)
sA1 <- na.omit(sA)
stA <- unique (sA1, incomparables =  FALSE)
sB <- ifelse(q1 <1001, as.character(mmmB$Gene.symbol), NA)
sB1 <- na.omit(sB)
stB <- unique (sB1, incomparables =  FALSE)
sC <- ifelse(q > zz-1001, as.character(mmmA$Gene.symbol), NA)
sC1 <- na.omit(sC)
stC <- unique (sC1, incomparables =  FALSE)
sD <- ifelse(q1 > zz-1001, as.character(mmmB$Gene.symbol), NA)
sD1 <- na.omit(sD)
stD <- unique (sD1, incomparables =  FALSE)
write.table (hA, file="pvalueA.txt", quote=F, sep="\t", col.names=FALSE, row.names=FALSE)
write.table (hB, file="pvalueB.txt", quote=F, sep="\t", col.names=FALSE, row.names=FALSE)
write.table (hC, file="pvalueC.txt", quote=F, sep="\t", col.names=FALSE, row.names=FALSE)
write.table (hD, file="pvalueD.txt", quote=F, sep="\t", col.names=FALSE, row.names=FALSE)
write.table (stA, file="namesA.txt", quote=F, sep="\t", col.names=FALSE, row.names=FALSE)
write.table (stB, file="namesB.txt", quote=F, sep="\t", col.names=FALSE, row.names=FALSE)
write.table (stC, file="namesC.txt", quote=F, sep="\t", col.names=FALSE, row.names=FALSE)
write.table (stD, file="namesD.txt", quote=F, sep="\t", col.names=FALSE, row.names=FALSE)
sA <- log10(hA)*-1
sB <- log10(hB)*-1
sC <- log10(hC)*-1
sD <- log10(hD)*-1
write.table (sA, file="log10A.txt", quote=F, sep="\t", col.names=FALSE, row.names=FALSE)
write.table (sB, file="log10B.txt", quote=F, sep="\t", col.names=FALSE, row.names=FALSE)
write.table (sC, file="log10C.txt", quote=F, sep="\t", col.names=FALSE, row.names=FALSE)
write.table (sD, file="log10D.txt", quote=F, sep="\t", col.names=FALSE, row.names=FALSE)
rrr <- dat2[ ,c(1,1,4,5,2,3)]
colnames(rrr) <- c("Unigene", "Gene Symbol", "r1","r2","metric1","metric2")
rrr2 <- rrr[order (rrr$r1),]
write.table(rrr2, file="tWITHz.txt", sep="\t",quote=F, col.names=TRUE, row.names=FALSE)
```
