# Supplementary material for: Creation of a gene expression portrait of depression and its application for identifying potential treatments
Source: Sci Rep. 2021 Feb 15;11:3829. doi: 10.1038/s41598-021-83348-0 (PMC7884719; doi:10.1038/s41598-021-83348-0)
Supplement: Supplementary file 2 — Supplementary Figures. [file 41598_2021_83348_MOESM2_ESM.pdf]

Supplementary Figures for:

Creation of a gene expression portrait of depression and its application for identifying potential treatments

Stephen C. Gammie
Department of Integrative Biology, University of Wisconsin-Madison, United States

Correspondence: scgammie@wisc.edu

Supplementary Figure 1. **Overview of steps involved in creating portraits of depression.** Left panel indicates human depression datasets used in the study, including Gene Expression Omnibus (GEO) numbers and brain region examined. Step 1: format each dataset to two columns, one for gene symbol (Gene.symbol) and the other (sign1) with data on direction of change in depression (positive = up in depression) and pvalue (-log10 transformation; higher absolute value of number reflects lower pvalue). Step 2: select datasets for portraits. Step 3: identify and rank genes consistently changed in the same direction in different datasets. Step 4: format portrait by gene symbol and information on direction of change and evidence for consistent change (sign1) to match the pattern for initial datasets. Abbreviations: ctx, cortex; pfc, prefrontal ctx, dlpfc, dorsolateral pfc; n. accum., nucleus accumbens; orb fr, orbitofrontal.

Supplementary Figure 2. **Comparison of portraits to individual datasets based on pvalues.** Pvalues for hypergeometric test are shown for each portrait when compared with a given dataset for genes moving in the same direction (A=up/up and D=down/down) and in opposite directions (B=up/down and C=down/up). Pvalues less than 1.0E-200 converted to zero. Gradual color scale ranges from 0 (red) to 1 (green). Column 'used' indicates with arrows whether that datasets was included in creation of the respective depression portraits. Relation of dataset identification to GEO study is provided in Supplementary Table 1.

Supplementary Figure 3. **Comparison of portraits to individual datasets based on overall scores.** Overall score for matching of portrait with a given dataset is based on –log10(pvalue) transform from hypergeometric test (higher positive value reflects lower pvalue). Column 'used' indicates with arrows whether that datasets was included in creation of the respective depression portraits. Overall score was derived by adding scores from same direction up/up and down/down) and subtracting scores from opposite directions (up/down and down/up). –log10(pvalue) of infinite converted to 400. Gradual color scale ranges from less than -5 (white) to 5 (green) to 800 (blue). Relation of dataset identification to GEO study is provided in Supplementary Table 1.

Supplementary Figure 4. **Overview of pathway for identifying and evaluating potential depression treatments.** Step 1: Create depression portraits that are based on CNS derived data (see above for details). Step 2: Enter top genes from portraits into drug repurposing tools to identify potential treatment candidates (note that datasets for repurposing are derived from multiple tissue and cell types and are not CNS specific). Step 3: Create a working list of potential treatments and add to this possible treatments (e.g., exercise) that are not found in drug repurposing tools. Step 4: Search GEO datasets for studies examining effect of treatment on CNS or related tissue (e.g., neuronal stem cells). Step 5: Create treatment datasets and format to be similar to those for portraits and allow for rank-rank comparisons. Step 6: Analyze treatments with portraits (note that both treatments and portraits are based on CNS expression). Step 7: Create output of treatment effects that allows for identification of top treatments.

Supplementary Figure 5. **Top potential new treatments for each portrait of depression.** Focusing on potential new antidepressant category, the top 25 treatments are shown for each portrait along with their overall rank and score. Similar color indicates same treatment, but different study or group comparisons. For example, exercise can reflect studies from humans or from mice exposed to running wheels. See Supplementary Table 2 for details on comparison made and study. Abbreviations are: a & g tocoph (alpha and gamma tocopherol with vitamin E); nic. riboside (nicotinamide riboside), insulin intran. (insulin intranasal); a linoic acid (alpha linoid acid).

Supplementary Fig. 1

Depression datasets

MEN ONLY

	GEO #	brain region
✓	GSE102556	insula ctx
✓	GSE102556	BA8 ctx
	GSE102556	BA11 ctx
	GSE102556	BA25 ctx
✓	GSE102556	n. accumb.
✓	GSE102556	hippocampus
✓	GSE54567	dlpfc
✓	GSE54572	cingulate ctx
✓	GSE54562	cingulate ctx
	GSE54565	cingulate ctx
✓	GSE53987	pfc
✓	GSE53987	hippocampus
✓	GSE53987	striatum
✓	GSE54566	amygdala
✓	GSE35978	cerebellum
✓	GSE35978	parietal ctx

WOMEN ONLY

✓	GSE102556	insula ctx
✓	GSE102556	BA8 ctx
	GSE102556	BA11 ctx
	GSE102556	BA25 ctx
✓	GSE102556	n. accumb.
✓	GSE102556	hippocampus
✓	GSE54568	dlpfc
✓	GSE54571	cingulate ctx
✓	GSE54563	cingulate ctx
✓	GSE53987	pfc
✓	GSE53987	hippocampus
✓	GSE53987	striatum
✓	GSE54564	amygdala
✓	GSE35978	cerebellum
✓	GSE35978	parietal ctx

MEN AND WOMEN

✓	GSE54570	dlpfc
✓	GSE54575	orb fr pfc
	GSE12654	BA10 ctx
✓	GSE101521	dlpfc

1. Differential gene expression for each dataset (depression v control)

Gene.symbol	sign1
DIRAS2	-3.08471
SEMA3E	-2.83483
SLC35F1	-2.66784
TMEM155	-2.37115
SSTR2	-2.36588
RETREG1	-2.34483
B3GALT2	-2.2163
SCD	-2.17741
RXFP1	-2.16379
SNORD59A	2.085204

sign1 indicates direction of change (negative = down, positive = up in depression) and significance (higher absolute value = greater significance)

steps: pvalue -log10 transformed, then multiplied by sign of change

2. Select datasets (avoid overrepresentation of same subjects)

✓ = used in depression portrait

combined portrait (men and women, all checked datasets)
male portrait (men only checked datasets)
female portrait (women only checked datasets)

3. Merge datasets and use rank information to identify genes consistently up or down. Genes up and down in different datasets are cancelled out. Steps of top 1000 up and down genes used with decreased weighting of lower significance genes.

4. Create portrait (e.g., combined portait)

Gene.symbol	sign1
EGR1	-17.8769
NR4A2	-15.6701
RPS23	14.76545
CH25H	-13.8988
EGR4	-13.6988
CRH	-13.5778
DUSP1	-13.5766
FOS	-13.5568
SPRY2	-13.4578
NR4A1	-13.3444
DUSP6	-12.7913

sign1 indicates direction of change (negative = down, positive = up in depression) and evidence (higher absolute value of number = higher net matches in given direction)

all genes ordered from most to least dysregulated

format matches original datasets (can be used for rank rank analysis)

Supplementary Fig. 2

Portraits compared with each dataset (pvalues)

		combined z1					male z2					female z3				
		same direction		opposite direction			same direction		opposite direction			same direction		opposite direction		
sex	dataset	A	D	B	C	used	A	D	B	C	used	A	D	B	C	used
female	x1	7.40E-59	2.67E-183	1	1	✓	6.98E-13	1.68E-57	0.637224	0.062844		4.66E-103	3.85E-231	1	1	✓
female	x2	7.00E-13	3.12E-45	0.47522	0.272734	✓	5.30E-01	4.83E-02	1.94E-40	4.12E-23		2.34E-49	1.99E-115	0.999995	1	✓
female	x3	7.35E-16	2.49E-82	0.931496	0.637409		3.61E-11	1.86E-17	7.22E-05	7.22E-05		1.17E-18	2.47E-116	0.752575	0.999912	
female	x4	3.41E-22	1.18E-74	0.980991	0.779821		1.57E-12	6.97E-10	0.001666	4.23E-08		5.32E-27	1.16E-105	0.989732	0.999978	
female	x5	5.36E-108	5.36E-108	1	1	✓	5.82E-30	4.02E-19	0.962344	1.52E-07		1.99E-115	9.38E-229	1	1	✓
female	x6	3.77E-56	3.99E-107	0.999725	0.999995	✓	1.04E-14	1.81E-25	9.10E-06	0.102133		3.03E-79	7.89E-157	1	1	✓
female	x7	4.66E-49	4.66E-49	1	0.999991	✓	1.03E-03	1.93E-07	0.000424	0.038019		2.90E-100	2.14E-71	1	1	✓
female	x8	8.72E-27	1.17E-20	1.00E+00	3.93E-08	✓	7.68E-01	3.94E-01	1.01E-04	2.56E-27		2.32E-81	4.54E-51	1.00E+00	1.00E+00	✓
female	x9	1.89E-21	1.89E-21	1	0.05505	✓	7.51E-02	5.70E-01	0.075114	6.15E-05		5.51E-69	3.39E-45	1	1	✓
female	x10	3.03E-35	2.70E-52	0.999999	1	✓	3.94E-01	1.01E-04	3.65E-05	0.000667		4.14E-101	7.62E-119	1	1	✓
female	x11	1.37E-10	8.92E-20	0.959707	0.999924	✓	9.81E-01	9.30E-01	9.82E-20	3.53E-12		4.50E-64	1.03E-44	1	1	✓
female	x12	5.01E-69	1.62E-37	1	0.999961	✓	3.78E-10	1.01E-04	0.066015	0.005163		3.03E-78	2.42E-85	1	1	✓
female	x13	2.51E-58	2.78E-33	1	1	✓	1.01E-04	1.47E-03	0.675082	0.995881		3.02E-68	3.44E-63	1	1	✓
female	x14	6.32E-54	2.32E-12	0.999994	1	✓	1.15E-03	9.86E-01	0.0519	0.803972		1.44E-77	8.11E-58	1	1	✓
female	x15	2.48E-37	8.76E-25	1	1	✓	2.94E-02	2.05E-01	0.99972	0.665065		1.70E-43	1.03E-62	1	1	✓
male	y1	5.61E-20	9.33E-35	0.779821	0.003639	✓	3.74E-56	2.53E-122	1	0.931432	✓	4.90E-01	7.32E-03	7.88E-34	2.11E-17	
male	y2	6.79E-97	1.08E-84	0.99422	1	✓	1.98E-213	7.07E-248	1	1	✓	3.68E-02	2.71E-04	1.46E-45	8.13E-18	
male	y3	4.34E-15	2.00E-27	9.96E-01	2.70E-21		2.56E-62	2.04E-72	1.00E+00	1.11E-03		1.97E-01	5.08E-03	4.36E-19	1.50E-24	
male	y4	1.18E-04	7.72E-11	1.87E-17	4.04E-19		7.69E-11	1.56E-05	1.04E-14	8.05E-08		5.08E-03	2.16E-07	1.07E-40	2.03E-12	
male	y5	1.87E-78	2.62E-138	1	1	✓	4.48E-150	7.72E-190	1	1	✓	4.03E-07	1.26E-36	0.01039	0.007316	
male	y6	2.11E-94	2.75E-67	0.986922	1	✓	1.24E-172	1.08E-130	1	1	✓	2.20E-10	3.09E-08	5.26E-15	4.32E-06	
male	y7	4.32E-38	5.39E-51	1	1	✓	2.65E-76	3.13E-112	1	1	✓	3.29E-06	3.25E-02	0.043391	0.094628	
male	y8	4.86E-59	3.00E-39	1	1	✓	2.09E-79	5.60E-71	1	1	✓	5.67E-09	2.19E-04	0.982649	0.119425	
male	y9	1.22E-55	6.37E-35	1	1	✓	2.42E-109	2.32E-92	1	1	✓	7.67E-02	1.23E-01	0.003116	0.225947	
male	y10	1.57E-05	4.98E-16	0.171226	0.875956		1.65E-04	1.36E-08	0.002371	0.962211		1.75E-05	5.82E-06	0.634363	0.012612	
male	y11	1.57E-49	6.35E-44	1	1	✓	3.16E-100	1.55E-84	1	1	✓	2.55E-04	9.62E-03	0.076173	0.034055	
male	y12	2.51E-29	5.09E-41	1	1	✓	2.60E-49	1.74E-69	1	1	✓	3.10E-01	2.99E-07	0.999797	0.008955	
male	y13	2.97E-39	7.84E-25	0.998546	1	✓	1.56E-75	5.00E-48	0.999943	1	✓	6.25E-03	6.34E-01	0.012612	0.073999	
male	y14	3.05E-35	1.30E-53	0.999924	0.999149	✓	4.30E-60	5.44E-74	1	1	✓	8.98E-03	1.85E-06	0.057225	0.074196	
male	y15	1.12E-15	1.52E-21	1	0.99967	✓	5.56E-23	1.83E-16	1	1	✓	2.36E-01	9.99E-03	0.490113	0.328374	
male	y16	3.69E-14	1.12E-17	1	1	✓	1.14E-15	5.97E-31	1	1	✓	6.02E-01	2.36E-01	1	0.96972	
both	xy1	3.63E-18	9.50E-37	0.999964	1	✓	1.32E-06	1.02E-04	0.444469	0.540904		8.02E-01	2.98E-02	1.54E-13	0.009629	
both	xy2	3.05E-15	4.92E-41	1	0.999998	✓	9.10E-01	2.40E-03	0.195701	0.092122		7.07E-03	7.21E-06	0.999891	0.029776	
both	xy3	0.02984	0.057099	0.569178	0.970305		0.007681	0.278336	0.473657	0.975648		4.48E-04	0.112316	0.995539	0.99841	
both	xy4	1.51E-101	6.25E-56	1	1	✓	3.29E-33	2.80E-06	1	0.972124		8.24E-10	6.52E-14	0.979508	0.944898	
combined	z1	0	0	1	1		0	0	1	1		1.86E-272	0	1	1	
male	z2	0	0	1	1		0	0	1	1		5.00E-05	7.02E-11	1.75E-05	0.000136	
female	z3	0	0	1	1		5.00E-05	7.02E-11	0.000136	1.75E-05		0	0	1	1	

A = up/up B = up/down C = down/up D = down/down

Supplementary Fig. 3

Portraits compared with each dataset (overall score)

		combined z1		male z2		female z3	
sex	dataset	score	used	score	used	score	used
female	x1	240.7039	✓	67.5336		332.7459	✓
female	x2	55.7735	✓	-60.5054		163.3329	✓
female	x3	96.5112		18.8913		133.4158	
female	x4	95.2805		10.8090		131.2050	
female	x5	214.5412	✓	40.7945		342.7296	✓
female	x6	161.8225	✓	32.6962		234.6215	✓
female	x7	96.6626	✓	4.9088		170.2067	✓
female	x8	38.5844	✓	-30.0663		130.9769	✓
female	x9	40.1885	✓	-3.9665		112.7290	✓
female	x10	86.0871	✓	-3.2153		218.5012	✓
female	x11	28.8941	✓	-30.4207		107.3332	✓
female	x12	105.0897	✓	9.9505		162.1361	✓
female	x13	90.1557	✓	6.6566		129.9831	✓
female	x14	64.8332	✓	1.5677		133.9339	✓
female	x15	60.6631	✓	2.0424		104.7565	✓
male	y1	50.7344	✓	176.9938	✓	-47.3325	
male	y2	180.1305	✓	459.8548	✓	-56.9238	
male	y3	20.4901		130.3264		-39.1840	
male	y4	-21.0810		-6.1567		-42.7018	
male	y5	215.3102	✓	338.4606	✓	38.1762	
male	y6	160.2293	✓	301.8711	✓	-2.4760	
male	y7	87.6331	✓	187.0807	✓	4.5832	
male	y8	96.8368	✓	148.9326	✓	10.9748	
male	y9	89.1105	✓	200.2513	✓	-1.1279	
male	y10	19.2820		9.0066		7.8949	
male	y11	92.0001	✓	183.3102	✓	3.0247	
male	y12	68.8932	✓	117.3450	✓	4.9849	
male	y13	62.6316	✓	122.1082	✓	-0.6285	
male	y14	87.4015	✓	132.6308	✓	5.4066	
male	y15	35.7711	✓	37.9925	✓	1.8349	
male	y16	30.3840	✓	45.1672	✓	0.8349	
both	xy1	53.4621	✓	9.2523		-13.2065	
both	xy2	54.8234	✓	0.9168		5.7666	
both	xy3	2.5107		2.3348		4.2961	
both	xy4	156.0255	✓	38.0230		22.2360	
combined	z1	800.0000		800.0000		671.7311	
male	z2	800.0000		800.0000		5.8298	
female	z3	671.7311		5.8298		800.0000	

Supplementary Fig. 4

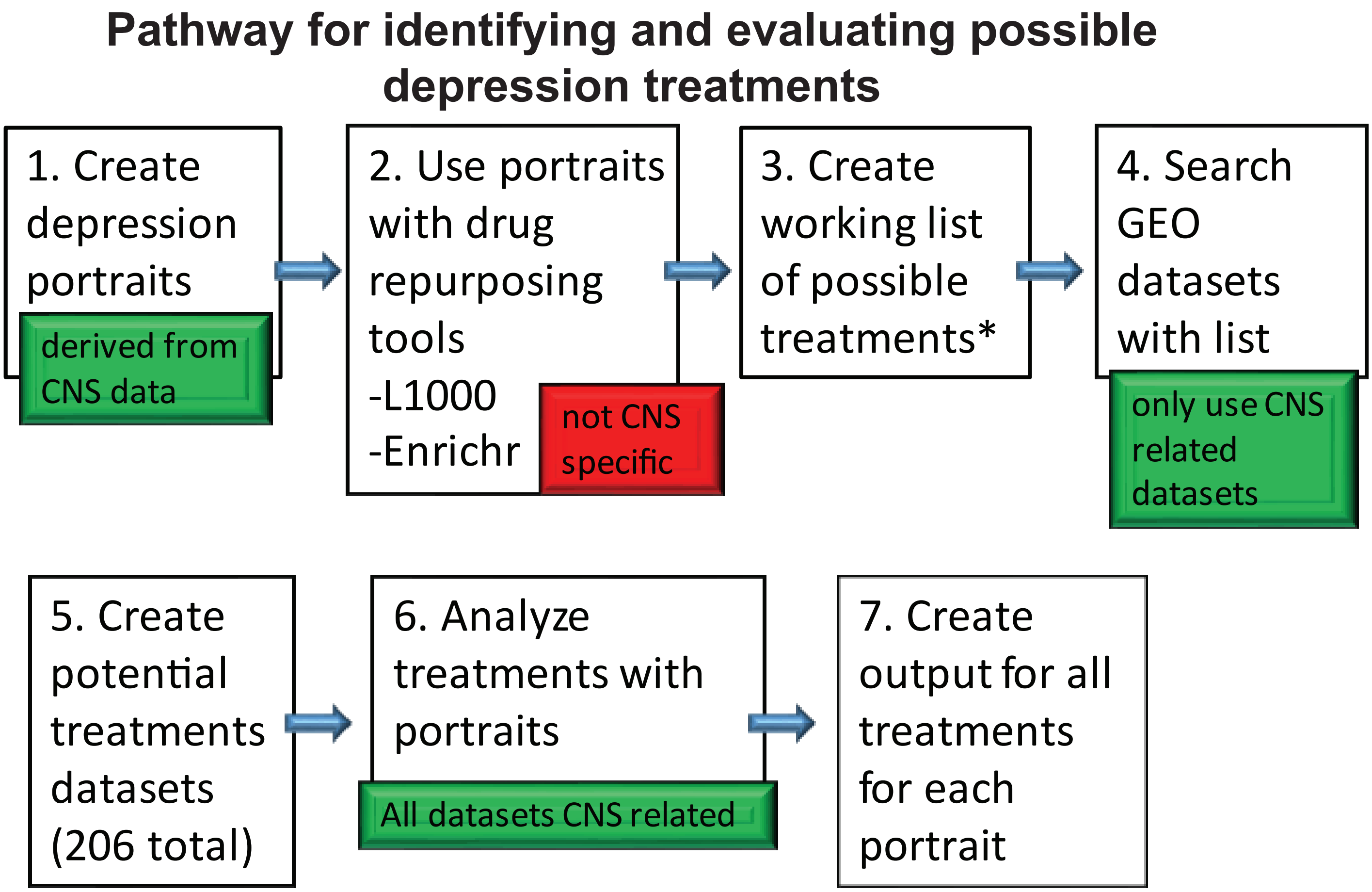

Supplementary Fig. 5

Top potential new treatments for each portrait

combined

rank	GEO #	treatment	score
1	GSE110298	exercise	37.924
2	GSE110298	exercise	23.285
9	GSE33137	curcumin	16.327
11	GSE128660	elevated temp.	14.007
25	GSE10748	D-serine 20 mg	9.978
29	GSE5140	creatine	8.7576
31	GSE8162	alpha & g toco.	8.5513
32	GSE109445	albiflorin	8.2755
33	GSE29075	exercise	8.084
36	GSE10748	D-serine 5 mg	7.5924
39	GSE62194	nicot. riboside	6.7451
41	GSE10748	D-serine 50 mg	6.366
48	GSE38465	exercise	5.3828
59	GSE124353	celastrol	3.6336
60	GSE20219	pioglitazone	3.5909
61	GSE20219	pioglitazone	3.5653
64	GSE62194	nicot. riboside	3.3064
68	GSE31430	zinc	3.2129
72	GSE20219	pioglitazone	3.0231
73	GSE20219	pioglitazone	3.0218

male

rank	GEO #	treatment	score
1	GSE110298	exercise	48.107
4	GSE33137	curcumin	24.026
10	GSE5140	creatine	16.256
13	GSE8162	alpha & g toco.	14.506
15	GSE110298	exercise	14.208
21	GSE109445	albiflorin	11.854
22	GSE29075	exercise	11.247
25	GSE62194	nicot. riboside	10.46
31	GSE10748	D-serine 20 mg	9.02
33	GSE10748	D-serine 5 mg	8.5465
34	GSE103031	aminolevulinic	8.4603
47	GSE10748	D-serine 50 mg	5.9439
50	GSE31430	zinc	5.5831
54	GSE84156	withaferin A	4.9606
56	GSE38465	exercise	4.7017
61	GSE109055	nicot. riboside	3.9749
73	GSE20219	pioglitazone	2.939
74	GSE20219	pioglitazone	2.9361
76	GSE62194	nicot. riboside	2.7434
77	GSE124353	celastrol	2.5294

female

rank	GEO #	treatment	score
2	GSE10748	D-serine 20 mg	16.08
3	GSE10748	D-serine 5 mg	14.082
15	GSE109445	albiflorin	7.9593
21	GSE10748	D-serine 50 mg	6.3348
24	GSE110298	exercise	6.1242
30	GSE62194	nicot. riboside	5.5924
34	GSE110298	exercise	5.1684
39	GSE62194	resveratrol	4.6422
43	GSE126996	exercise	4.2619
46	GSE33137	curcumin	3.8159
47	GSE110298	exercise	3.5843
51	GSE38465	exercise	3.2683
58	GSE32536	pioglitazone	2.9118
59	GSE32536	pioglitazone	2.9118
61	GSE62194	nicot. riboside	2.6197
62	GSE61326	vitamin D	2.5631
66	GSE130099	insulin intran.	2.2166
76	GSE10748	D-serine 500 mg	1.7625
77	GSE29075	exercise	1.6238
85	GSE20219	pioglitazone	1.152
